# Supplementary material for: Impacts of Brexit on fruit and vegetable intake and cardiovascular disease in England: a modelling study
Source: BMJ Open. 2019 Jan 28;9(1):e026966. doi: 10.1136/bmjopen-2018-026966 (PMC6352794; doi:10.1136/bmjopen-2018-026966)
Supplement: Supplementary file 1 [file bmjopen-2018-026966supp001.pdf]

Supplementary Appendix

Appendix A. Trade data inputs

The data inputs used in the policy layer of the model are presented in Table A1. These include fruit and vegetable (F&V) imports to the UK from the EU and from non-EU countries, either dutiable or under a duty-free arrangement, estimates of applied import tariffs, and estimates of imports as a percentage of overall F&V supply. We used a combination of data from the World Trade Organization (WTO),[1] the HM Revenues and Customs (HMRC),[2] and the Department for Environment, Food & Rural Affairs (DEFRA)[3] to obtain these estimates. The methodology used is described in detail below.

F&V were classified into five groups of fruits and seven groups of vegetables, in accordance to the classification applied by Tiffin et al. to estimate the price elasticities used in this model. We also identified two categories of canned, dried, and frozen F&V that were used in the sensitivity analysis. We used the Harmonised System (HS) 2012 classification to assign traded commodities to these groups. The HS has been developed by the World Customs Organization and has international applications, facilitating trade across participating countries. It uses a six-digit code system, arranged in three levels of disaggregation (two-digit, four-digit, and six-digit) to classify commodities. In this analysis, we used the four-digit HS level. The commodities included in each subgroup of F&V are presented in Table A2.

We used the latest available data (2015) from the HMRC[2] to identify imports of F&V (in net mass, kg) to the UK by country of origin for each F&V subgroup. Using information from the WTO, we identified the duty arrangement that is in place between each of the UK's F&V importers and the EU. These included bilateral or multilateral preferential arrangements or, if no agreement was in place, trade under the Most Favoured Nation (MFN) principle. The MFN principle prevents WTO members from discriminating between countries by imposing a common MFN duty to all their trading partners that are not part of a preferential trade agreement. We, then, identified which preferential arrangements allowed for duty-free trade for each F&V subgroup. As different duty rates are applied to the single products that comprise each commodity group (tariff lines), we defined as a duty-free arrangement every arrangement that gave duty-free access to at least 50% of tariff lines in each F&V subgroup.

We used data from the WTO[1] to estimate the mean ad valorem MFN duty applied by the EU and its standard deviation for each F&V subgroup. The WTO provides several averaging methods for applied tariffs in each commodity group. We used the HS sub-heading averaging method which gives equal importance to all HS sub-headings included in each commodity group.[1] The standard

deviation shows the dispersion of HS sub-heading averages around the overall simple average of the commodity group. This method is not biased by the different levels of disaggregation in the applied tariffs across WTO members, allowing for a uniform comparison across countries. When more than one commodity code was included in our F&V subgroups (i.e. for other and canned, dried, and frozen F&V), we used the same method to average across groups by summing the average tariff of each commodity code and dividing by the total number of subheadings included in the group. The combined standard deviation for these groups was estimated using the following formula:

$$Combined\ SD = \frac{\sqrt{\sum(n*(s^2+(\bar{x}-\bar{X})^2)}}{\sum n}$$

where n is the number of subheadings, s the standard deviation for each subheading, $\bar{x}$ the average tariff for each subheading, and $\bar{X}$ the average tariff of all subheadings.

Tariffs for all F&V subgroups were ad valorem with the exception of tomatoes which have a specific duty defined in €/100 kg. The World Bank provides estimations of the ad valorem equivalent of specific duties.[4] These varied based on the declared price of tomatoes. We conservatively used the smaller tariff provided, as tomato price is seasonal and fluctuates significantly thoughtout the year.

Finally, we estimated F&V imports as a percentage of F&V supply. DEFRA provides information on imports, exports, and production of F&V.[3] Total supply was defined as the sum of domestic production and imports minus exports. We estimated imports as a percentage of total supply for years 2006 to 2016 to capture annual variations at the medium term. We then used the mean and standard deviation of these estimates for our model. We assigned the F&V products provided by DEFRA into our F&V subgroups as shown in Table A2.

Table A1. Trade related inputs of the model policy layer.

	Imports from the EU (% of total imports)	Duty-free imports from non-EU countries (% of total imports)	Dutiable imports from non-EU countries (% of total imports)	Ad valorem EU MFN duty (%) Mean (std dev)	Imports (% of supply) Mean (std dev)
Fruits					
Apples and Pears	67.1	21.5	11.4	7.2 (0.0)	71.5 (2.4)
Bananas	10.2	89.2	0.6	16.0 (0.0)	100.0 (0)
Citrus fruit	51.2	43.1	5.7	10.8 (5.5)	100.0 (0)
Grapes	26.2	63.8	10.1	8.4 (6.0)	100.0 (0)
Other Fruits	49.2	38.2	12.6	7.0 (3.4)	87.0 (1.2)
Vegetables					
Brassica	97.0	2.7	0.3	12.0 (0.0)	31.0 (4.6)
Legumes	18.7	78.5	2.8	9.6 (1.6)	92.6 (8.2)
Lettuce	99.8	0.1	0.0	10.4 (0.0)	58.8 (4.1)
Onions	82.4	8.5	9.2	10.0 (0.4)	50.9 (2.5)
Other vegetables	88.5	9.0	2.5	9.8 (3.5)	70.6 (2.1)
Root vegetables	86.2	13.4	0.3	13.3 (0.3)	5.1 (1.3)
Tomatoes	88.6	0.3	11.2	10.8 (0.0)	82.8 (1.2)
Canned, dried, and frozen					
Fruits	61.7	22.6	15.7	8.1 (5.2)	87.0 (1.2)
Vegetables	62.5	7.2	30.3	7.3 (6.8)	70.6 (2.1)

Table A2. Classification of F&V across different data sources.

	Trade and Tariff data (HS 4-digit classification)	Supply data (DEFRA)	Living Costs and Food Survey – household purchases
Fruits			
Apples and Pears	0808 - Apples, pears and quinces, fresh	Total Dessert Apples; Total Culinary Apples; Total Pears	Fresh apples, Fresh pears
Bananas	0803 - Bananas, incl. plantains, fresh or dried	Bananas	Fresh bananas
Citrus fruit	0805 - Citrus fruit, fresh or dried	Citrus	Fresh oranges, Other fresh citrus fruits
Grapes	0806 - Grapes, fresh or dried	Grapes	Fresh grapes

Other Fruits	0804 - Dates, figs, pineapples, avocados, guavas, mangoes and mangosteens, fresh or dried; 0807 - Melons, incl. watermelons, and papaws "papayas", fresh; 0809 - Apricots, cherries, peaches incl. nectarines, plums and sloes, fresh; 0810 - Fresh strawberries, raspberries, blackberries, back, white or red currants, gooseberries and other edible fruits	Total Plums; Cherries; Others & Mixed; Strawberries; Raspberries; Blackcurrants; Other Soft Fruit	Fresh stone fruit, Other fresh soft fruit, Fresh melons, Other fresh fruit
Vegetables			
Brassica	0704 - Cabbages, cauliflowers, kohlrabi, kale and similar edible brassicas, fresh or chilled	Cauliflowers and Broccoli; Cabbages; Other Brassicas	Fresh cabbages, Fresh brussels sprouts, Fresh cauliflower
Legumes	0708 - Leguminous vegetables, shelled or unshelled, fresh or chilled	Beans; Peas; Dried Peas; Dried Chick Peas; Dried Beans	Fresh peas, Fresh beans
Lettuce	0705 - Lettuce "Lactuca sativa" and chicory "Cichorium spp.", fresh or chilled	Lettuce; Lettuce - protected veg	Leafy salads fresh (Lettuce and leafy salads, Prepared lettuce salads)
Onions	0703 - Onions, shallots, garlic, leeks and other alliaceous vegetables, fresh or chilled	Onions, Dry Bulb; Onions, Spring; Leeks; Garlic; Other Alliaceous; Onions	Fresh onions, leeks and shallots
Other vegetables	0707 - Cucumbers and gherkins, fresh or chilled; 0709 - Other vegetables, fresh or chilled	Asparagus; Baby leaf; Celery; Courgette; Cucumbers; Mushrooms; Others-protected veg; Others, field grown; Rhubarb; Self Blanching Celery; Sweet Peppers; Watercress; All Other Dried Vegetables; All Other Fresh Vegetables; Aubergines	Other fresh green vegetables, Fresh cucumbers, Fresh mushrooms, Miscellaneous fresh vegetables
Root vegetables	0706 - Carrots, turnips, salad beetroot, salsify, celeriac,	Beetroot; Carrots; Parsnips; Turnips and	Fresh carrots, Fresh turnips and swede, Other fresh root vegetables

	radishes and similar edible roots, fresh or chilled	Swedes; Carrots and Turnips	
Tomatoes	0702 - Tomatoes, fresh or chilled	Tomatoes	Fresh tomatoes
	Canned, dried, and frozen		
Fruits	0811 - Fruit and nuts, uncooked or cooked by steaming or boiling in water, frozen, whether or not containing added sugar or other sweetening matter; 0813 - Dried apricots, prunes, apples, peaches, pears, papaws "papayas", tamarinds and other edible fruits, and mixtures of edible and dried fruits or of edible nuts; 0814 - Peel of citrus fruit or melons, incl. watermelons, fresh, frozen, dried or provisionally preserved in brine, or in water with other additives	Approximated from "Other Fruits"	Tinned peaches, pears and pineapples; All other tinned or bottled fruit; Dried fruit; Frozen strawberries, apple slices, peach halves, oranges and other frozen fruits
Vegetables	0710 - Vegetables, uncooked or cooked by steaming or boiling in water, frozen; 0712 - Dried vegetables, whole, cut, sliced, broken or in powder, but not further prepared 0713 - Dried leguminous vegetables, shelled, whether or not skinned or split	Approximated from "Other Vegetables"	Tomatoes, canned or bottled; Peas, canned; Beans canned; Other canned vegetables; Dried pulses other than air-dried; Air-dried vegetables; Peas, frozen; Beans, frozen; Other frozen vegetables

Appendix B. Mortality projections.

We used a Bayesian Age-Period-Cohort (BAPC) model to estimate mortality projections between 2021 and 2030. The BAPC model attributes changes in mortality to (1) age effects, which capture physiological and social changes related to aging; (2) period effects, which are associated with environmental, scientific, and social changes related to calendar time of death; and (3) cohort effects, which describe generational differences due to year of birth. It then projects mortality probabilities by assuming that trends of these effects continue over time. The methodology has been previously described in detail.[5] Briefly, we used historic CHD and stroke mortality data stratified by sex and age (10-year groups from 25-34 until 85+) between 1981 and 2015 from the Office for National Statistics (ONS), defined using ICD-codes (Table B1). We also used historic population estimates (1985-2015) and population projections (2016-2030) by age and sex from the ONS. We applied the model using the BAMP statistical software which employs Markov Chain Monte Carlo simulations to estimate mortality projections. An age and sex specific ischemic to haemorrhagic stroke ratio (Table B2), estimated using 2016 mortality data by ICD-code from the ONS, was applied to overall stroke projections to estimate ischemic and haemorrhagic stroke mortality between 2021 and 2030, assuming no changes in clinical care over time. We combined ischemic and not specified stroke to better reflect evidence from the American Heart Association that suggests 87% of all stroke is ischemic.[6] Previous analyses in the UK have followed the same approach.[7]

Table B1. ICD codes for CVD outcomes

Type of CVD outcomes	ICD-9 codes (1981-2000)	ICD-10 codes (2011-2015 and 2016)
CHD	410-414	I20-I25
Overall stroke	430-438	I60-I69
Ischemic stroke		I63, I65-I67 (except I67.4)
Haemorrhagic stroke		I60-I62, I69.0-I69.2, I67.4
Other (not specified) stroke		I64, I69.4, I69.8

Table B2. Percentage of stroke deaths attributed to ischemic, haemorrhagic, and other (not specified) stroke and ischemic to haemorrhagic stroke ratio

Population group	Ischemic stroke	Haemorrhagic stroke	Other (not specified) stroke	Ischemic: Haemorrhagic*
Men 25-34	14%	82%	5%	0.22
Men 35-44	12%	79%	9%	0.26
Men 45-54	17%	67%	16%	0.50
Men 55-64	18%	53%	30%	0.90
Men 65-74	23%	37%	40%	1.71
Men 75-84	24%	28%	48%	2.57
Men 85+	30%	19%	51%	4.25
Women 25-34	23%	69%	9%	0.46
Women 35-44	14%	81%	5%	0.23
Women 45-54	14%	73%	13%	0.38
Women 55-64	15%	63%	23%	0.59
Women 65-74	18%	46%	36%	1.16
Women 75-84	23%	32%	46%	2.13
Women 85+	30%	17%	53%	4.98
Overall	*26%*	*28%*	*46%*	*2.58*

*Other (not specified) stroke was combined with ischemic stroke.

Appendix C. Effect of Brexit scenarios on price of F&V

Under each scenario, we assumed two main drivers of F&V price change. The first driver is the application of tariffs to imported commodities based on their country of origin. If there is a free trading agreement in place, these tariffs will be zero. However, if trade occurs under WTO regulations, the UK will have to apply import tariffs. These are required to be equal among all trading partners under the WTO's MFN clause that protects its members from discriminatory treatment. We assumed that the UK will adopt the EU MFN tariffs for all scenarios except scenario 4 (Liberalised regime) when the UK will reduce its MFN tariffs to zero.

The second driver of price change is attributed to an increase in transaction costs of EU trade due to post-Brexit border controls. We assumed that border controls will occur (1) to apply rules of origin and (2) to check technical requirements, such as Technical Barriers to Trade (TBT) and Sanitary and Phytosanitary (SPS) measures. Rules of origin determine commodities' country of origin and are necessary in order to apply appropriate trade restrictions such as tariffs. Within the Customs Union, such restrictions do not apply and goods move across borders freely. However, exiting the EU Customs Union will require checks for rules of origin for all commodities entering the UK from the EU. The cost of ensuring compliance with rules of origin regulations varies between different countries, depending on their trade arrangements, their infrastructure, the product traded, and other factors[8]. A WTO Working Paper[8] suggests that administrative costs of compliance with rules of origin vary between 2% and 8%, recommending a 5% estimate as a commonly used benchmark.

In common with other free trading arrangements, the EU has integrated technical regulations that do not require border controls. However, when no free trading agreement is in place and trade occurs under WTO regulations, technical characteristics of commodities need to be checked at the border. Cadot et al. investigated the difference between the price-raising effect of SPS and TBT by commodity in the presence and absence of preferential arrangements that involve deep integration of technical requirements[9]. They estimated a difference of 4.5% for vegetable products, which we used to approximate the increase in transaction costs of F&V under the Brexit scenarios that no preferential arrangement is assumed between the UK and the EU (Scenario 3 – No deal Brexit and Scenario 4 – Liberalised regime). However, this might overestimate the real effect as the analysis by Cadot et al. does not specify if the price-raising effects of SPS and TBT are due to transaction costs or demand increases reflecting the quality improvements that usually follow compliance with technical requirements.

Appendix D. Estimating the effect of F&V intake change on mortality

The IMPACT Food Policy model estimates the proportional change in mortality due to change in the intake of each F&V subgroup by age and sex using the following formula:

$$MortalityChange = (1 - e^{beta \times IntakeChange})$$

where *beta* is the natural logarithm of the relative risk between fruit or vegetable intake and CVD outcomes and *IntakeChange* is the change in intake of each F&V subgroup under the different scenarios.

The number of deaths attributable to each scenario for every F&V subgroup by age, sex, and year are then estimated as shown below:

$$Deaths = MortalityChange * Mortality$$

where *Mortality* is the counterfactual number of predicted deaths of CHD, ischemic stroke or haemorrhagic in each age-sex group in years 2021 to 2030.

In order to combine the effect of the different F&V subgroups into an overall F&V effect we used the cumulative risk-reduction approach as previously described by Bajekal et al.[10] Briefly, we estimated an adjustment factor as the ratio of an additive and a multiplicative effect as shown below:

$$AF = CR/AR$$

where

$$CR = 1 - \big(1 - abs(MortalityChange_1)\big) * \big(1 - abs(MortalityChange_2)\big) * \dots * \big(1 - abs(MortalityChange_i)\big)$$

and

$$AR = abs(MortalityChange_1) + abs(MortalityChange_2) + \cdots + abs(MortalityChange_i)$$

with *1, 2, …, i* being the different F&V subgroups.

We then estimated the overall attributable deaths as shown in the following equation

$$Deaths_{overall} = (Deaths_1 + Deaths_2 + \cdots + Deaths_i) * AF$$

where $Deaths_1$, $Deaths_2$, …, $Deaths_i$ are the number of deaths due to change in intake of each F&V subgroup.

Appendix E. Sensitivity analysis – substitution with canned, dried, and frozen F&V

We performed a sensitivity analysis in order to take into account substitution between fresh F&V and canned, dried, and frozen F&V. Trade data inputs for canned, dried, and frozen F&V and their classification using commodity codes are presented in Tables A1 and A2 respectively. Intake of canned, dried, and frozen F&V was estimated by applying a gradient of purchases from the Living Costs and Food Survey[11] to the overall F&V intake, similarly to F&V subgroups estimation (Table A2). We used own and cross-price elasticities for "Fresh fruits", "Fresh vegetables", "Tinned & dried fruit", and "Canned vegetables" by Tiffin et al. Frozen F&V were not included in the estimation of price elasticities.

For the model calculations, we first estimated the cross-price effect of the change in the price of fresh F&V on canned, dried, and frozen F&V using the appropriate cross-price elasticity. The effect of the different Brexit scenarios on price of overall fresh F&V was calculated as the ratio of the final changes in fresh F&V intake (after substitution between F&V subgroups was taken into account) to the own-price elasticities of fresh fruits and fresh vegetables.

We, then, estimated the effect of the different Brexit scenarios on prices of canned, dried, and frozen F&V, as described in Appendix B. Own and cross-price elasticities were employed to estimate the effect of the change in the price of canned, dried, and frozen F&V on the intake of canned, dried, and frozen F&V (own-price effect) and fresh F&V (cross-price effect), after the new equilibrium was reached. The overall intake change was the difference between baseline intake and intake when both prices of fresh F&V and canned, dried, and frozen F&V had changed. Effect of change in intake of fresh F&V on mortality was estimated as described in Appendix C.

Supplementary tables

Supplementary Table 1. Relative risks of the association between F&V intake and CHD, ischemic stroke and haemorrhagic stroke.

	RR per serving of fruit intake			RR per serving of vegetable intake		
Age group	CHD	Ischemic stroke	Haemorrhagic stroke	CHD	Ischemic stroke	Haemorrhagic stroke
25-34	0.92 (0.87, 0.97)	0.83 (0.76, 0.9)	0.63 (0.49, 0.81)	0.93 (0.89, 0.97)	0.76 (0.64, 0.9)	0.76 (0.61, 0.95)
35-44	0.92 (0.87, 0.97)	0.83 (0.77, 0.9)	0.64 (0.5, 0.82)	0.93 (0.9, 0.97)	0.77 (0.66, 0.9)	0.77 (0.62, 0.95)
45-54	0.93 (0.89, 0.97)	0.86 (0.8, 0.92)	0.69 (0.56, 0.84)	0.94 (0.91, 0.97)	0.80 (0.7, 0.92)	0.80 (0.67, 0.96)
55-64	0.94 (0.91, 0.98)	0.88 (0.83, 0.93)	0.73 (0.61, 0.87)	0.95 (0.93, 0.98)	0.83 (0.74, 0.93)	0.83 (0.72, 0.96)
65-74	0.95 (0.92, 0.98)	0.90 (0.86, 0.94)	0.77 (0.67, 0.89)	0.96 (0.94, 0.98)	0.86 (0.78, 0.94)	0.86 (0.76, 0.97)
75+	0.97 (0.96, 0.99)	0.94 (0.92, 0.96)	0.86 (0.8, 0.92)	0.98 (0.97, 0.99)	0.92 (0.87, 0.96)	0.92 (0.86, 0.97)

Source: Micha et al, 2017[12]

Supplementary Table 2. Statistical distributions for model inputs and corresponding parameters used in the probabilistic sensitivity analysis.

Inputs	Distribution	Parameters	Source
Ad valorem EU MFN tariff (%)	Beta	Alpha=((1-mean)/variance - 1/mean)*mean^2; beta=alpha*(1/mean - 1) estimated from tariff averages and variance	Tariff Analysis Online, WTO[1]
Imports % supply	Beta	Alpha=((1-mean)/variance - 1/mean)*mean^2; beta=alpha*(1/mean - 1) estimated from the mean and variance of imports between 2006-2016	DEFRA[3]
Cost of rules of origin	Pert	mode: 5%; min: 2%; max: 8%	Abreu, 2013[8]
Cost of technical regulations	Pert	mode: 4.5%, max/min: +/-20%	Cadot, 2016[9]
Mortality	Pert	mode: death projections, best estimate; min: death projections, lower 95% credible limit; max: death projections, upper 95% credible limit	Own estimations using ONS data
Elasticities	Normal	Mean and SE using price elasticities between 2000 and 2009	Tiffin, 2011[13]
F&V intake	Gamma	alpha: mean^2/variance; beta: variance/mean	National Diet and Nutrition Survey Rolling Programme[14]
Relative Risks	Log normal	Parameters based on Barendregt, 2010[15]	Micha, 2017[12]

Supplementary Table 3. Estimated relative change in price of fruit and vegetable subgroups under each modelled Brexit scenario. Numbers in parentheses indicate 95% uncertainty intervals.

	Scenario 1	Scenario 2	Scenario 3	Scenario 4
Fruits				
Apples and Pears	2.9% (2.0%, 4.0%)	3.5% (2.5%, 4.6%)	9.1% (8.0%, 10.4%)	4.0% (3.0%, 5.1%)
Bananas	7.6% (7.4%, 7.9%)	14.8% (14.6%, 15.0%)	16.9% (16.6%, 17.1%)	0.9% (0.6%, 1.1%)
Citrus fruit	4.7% (2.7%, 7.8%)	6.8% (3.5%, 12.9%)	14.3% (7.6%, 27.2%)	4.2% (3.0%, 5.6%)
Grapes	3.5% (1.4%, 9.1%)	5.8% (1.7%, 16.9%)	8.7% (3.1%, 24.3%)	1.8% (0.1%, 2.7%)
Other fruits	3.2% (2.1%, 4.9%)	4.3% (2.5%, 7.3%)	9.0% (5.4%, 15.7%)	3.3% (2.1%, 4.4%)
Vegetables				
Brassica	1.5% (0.8%, 2.4%)	1.6% (0.8%, 2.5%)	6.5% (4.5%, 8.7%)	2.8% (1.8%, 4.1%)
Legumes	4.4% (3.0%, 5.9%)	7.8% (5.4%, 10.8%)	10.3% (7.1%, 13.9%)	1.4% (1.0%, 1.9%)
Lettuce	2.9% (1.7%, 4.3%)	2.9% (1.7%, 4.3%)	11.6% (9.6%, 13.9%)	5.5% (4.1%, 7.2%)
Onions	2.3% (1.4%, 3.3%)	2.5% (1.6%, 3.5%)	8.6% (7.4%, 9.9%)	3.5% (2.5%, 4.5%)
Other vegetables	3.4% (2.1%, 4.8%)	3.7% (2.3%, 5.1%)	12.3% (8.2%, 18.0%)	5.7% (4.3%, 7.2%)
Root vegetables	0.3% (0.1%, 0.5%)	0.3% (0.2%, 0.5%)	1.1% (0.6%, 1.7%)	0.4% (0.2%, 0.7%)
Tomatoes	3.8% (2.1%, 5.3%)	3.8% (2.1%, 5.3%)	14.9% (13.2%, 16.6%)	6.0% (4.4%, 7.7%)

Supplementary Table 4. Mean (standard deviation) intake of F&V (g/d) in England by age and sex, estimated using data from the National Diet and Nutrition Survey Rolling Programme Years 1-8.

Population group	Fruit intake	Vegetable intake
Men 25-44	86.5 (95.3)	187.9 (99.3)
Men 45-64	116.6 (114.0)	200.7 (109.0)
Men 65+	121.6 (111.8)	184.6 (101.8)
Women 25-44	93.3 (102.1)	184.8 (111.6)
Women 45-64	128.5 (118.1)	202.0 (115.0)
Women 65+	132.2 (117.1)	173.6 (86.3)
Overall	*111.1 (111.1)*	*190.0 (106.1)*

Means and standard deviation are weighted for non-response

Supplementary Table 5. Gradient of F&V subgroups intake, estimated using household purchase data (g/person/week) from the Family Food module of the Living Costs and Food Survey 2016/7.

F&V subgroup	Intake (%)
Apples & Pears	19.1
Bananas	25.5
Citrus fruit	16.1
Grapes	6.8
Other fruits	26.9
Canned, dried, and frozen fruits	*5.5*
Total fruits	***100.0***
Brassica	9.4
Legumes	2.0
Lettuce	4.5
Onions	10.4
Other vegetables	24.6
Root vegetables (excl. potatoes)	16.6
Tomatoes	7.7
Canned, dried, and frozen vegetables	*24.8*
Total vegetables	***100.0***

Supplementary Table 6. Estimated relative change in intake of F&V subgroups under each modelled Brexit scenario. Numbers in parentheses indicate 95% uncertainty intervals.

	Scenario 1	Scenario 2	Scenario 3	Scenario 4
Fruits	***-4.4% (-5.2%, -3.8%)***	***-7.0% (-8.4%, -5.9%)***	***-11.4% (-14.2%, -9.5%)***	***-2.8% (-3.3%, -2.3%)***
Apples and Pears	-3.3% (-4.1%, -2.5%)	-4.4% (-5.4%, -3.5%)	-9.4% (-10.7%, -8.2%)	-3.5% (-4.3%, -2.7%)
Bananas	-5.4% (-5.8%, -5.0%)	-10% (-10.8%, -9.3%)	-12.1% (-13.2%, -11.2%)	-1.1% (-1.4%, -0.9%)
Citrus fruit	-4.4% (-7.1%, -2.7%)	-6.4% (-11.6%, -3.6%)	-13.0% (-24%, -7.3%)	-3.8% (-5%, -2.7%)
Grapes	-3.3% (-6.7%, -2.0%)	-5.4% (-11.9%, -3.0%)	-8.2% (-17.3%, -4.9%)	-1.8% (-2.4%, -0.7%)
Other fruits	-4.3% (-5.7%, -3.3%)	-6.2% (-8.7%, -4.7%)	-11.4% (-16.8%, -8.3%)	-3.6% (-4.6%, -2.5%)
Vegetables	***-2.5% (-3.1%, -1.9%)***	***-2.7% (-3.3%, -2.2%)***	***-9.1% (-11.0%, -7.8%)***	***-4.0% (-4.6%, -3.4%)***
Brassica	-1.5% (-2.2%, -1.0%)	-1.5% (-2.2%, -0.9%)	-6.4% (-8.1%, -4.9%)	-2.8% (-3.7%, -2.0%)
Legumes	-3.3% (-4.5%, -2.3%)	-5.7% (-7.8%, -4.0%)	-8.3% (-11%, -6.1%)	-1.6% (-2.1%, -1.1%)
Lettuce	-2.7% (-3.7%, -1.8%)	-2.9% (-3.9%, -2.0%)	-10.2% (-11.8%, -8.7%)	-4.6% (-5.9%, -3.6%)
Onions	-2.2% (-3%, -1.5%)	-2.4% (-3.2%, -1.7%)	-8.3% (-9.5%, -7.3%)	-3.5% (-4.3%, -2.7%)
Other vegetables	-3.7% (-5.0%, -2.6%)	-4.1% (-5.5%, -2.9%)	-13.4% (-18.6%, -9.7%)	-6.1% (-7.5%, -4.9%)
Root vegetables	-0.9% (-1.1%, -0.6%)	-0.9% (-1.2%, -0.6%)	-3.5% (-4.2%, -2.8%)	-1.5% (-1.8%, -1.2%)
Tomatoes	-3% (-4.1%, -1.9%)	-3.1% (-4.2%, -2%)	-11.8% (-13.1%, -10.6%)	-4.8% (-5.9%, -3.6%)

Supplementary Table 7. Estimated number of counterfactual (95% credible interval) and attributable (95% uncertainty interval) CHD and stroke deaths and mortality increase (%) in 2030, by age and sex, under each modelled Brexit scenario.

		Scenario 1	Scenario 2	Scenario 3	Scenario 4
CVD	Counterfactual deaths (2030)	71120 (38140, 164210)	71120 (38140, 164210)	71120 (38140, 164210)	71120 (38140, 164210)
	Attributable deaths (2030)	450 (250, 810)	630 (340, 1,180)	1,340 (780, 2,350)	450 (270, 750)
	Mortality reduction (%)	0.6%	0.9%	1.9%	0.6%
CHD	Counterfactual deaths (2030)	43110 (27160, 73740)	43110 (27160, 73740)	43110 (27160, 73740)	43110 (27160, 73740)
	Attributable deaths (2030)	140 (80, 230)	200 (110, 340)	420 (260, 690)	140 (90, 210)
	Mortality reduction (%)	0.3%	0.5%	1.0%	0.3%
Stroke	Counterfactual deaths (2030)	28010 (10970, 90470)	28010 (10970, 90470)	28010 (10970, 90470)	28010 (10970, 90470)
	Attributable deaths (2030)	320 (180, 590)	440 (240, 860)	960 (550, 1720)	330 (190, 570)
	Mortality reduction (%)	1.1%	1.5%	3.3%	1.1%

Supplementary Table 8. Estimated relative change in price of fresh and canned, dried, and frozen F&V under each modelled Brexit scenario. Numbers in parentheses indicate 95% uncertainty intervals.

	Scenario 1	Scenario 2	Scenario 3	Scenario 4
Fresh fruits	4.4%<br>(3.8%, 5.2%)	7.1%<br>(6%, 8.5%)	11.6%<br>(9.6%, 14.4%)	2.9%<br>(2.4%, 3.3%)
Canned, dried, and frozen fruits	3.4%<br>(2.1%, 5%)	4.1%<br>(2.4%, 6.9%)	10.3%<br>(5.7%, 20.0%)	4%<br>(2%, 5.6%)
Fresh vegetables	2.5%<br>(2%, 3%)	2.7%<br>(2.2%, 3.3%)	9.2%<br>(7.8%, 11%)	4%<br>(3.4%, 4.6%)
Canned, dried, and frozen vegetables	2.4%<br>(1.4%, 3.3%)	2.6%<br>(1.5%, 3.9%)	6.8%<br>(3.9%, 16.7%)	3.1%<br>(-1.2%, 4.8%)

Supplementary Table 9. Estimated relative change in intake of fresh and canned, dried, and frozen F&V under each modelled Brexit scenario. Numbers in parentheses indicate 95% uncertainty intervals.

	Scenario 1	Scenario 2	Scenario 3	Scenario 4
Fresh fruits	-4.5%<br>(-5.3%, -3.8%)	-7.1%<br>(-8.5%, -6.0%)	-11.7%<br>(-14.4%, -9.7%)	-2.9%<br>(-3.4%, -2.4%)
Canned, dried, and frozen fruits	-2.4%<br>(-3.7%, -1.3%)	-2.8%<br>(-5.0%, -1.3%)	-7.3%<br>(-15.3%, -3.8%)	-3.0%<br>(-4.2%, -1.4%)
Fresh vegetables	-2.6%<br>(-3.2%, -2.1%)	-2.9%<br>(-3.5%, -2.3%)	-9.6%<br>(-11.4%, -8.2%)	-4.1%<br>(-4.8%, -3.6%)
Canned, dried, and frozen vegetables	-1.0%<br>(-1.6%, -0.4%)	-1.1%<br>(-1.9%, -0.4%)	-2.6%<br>(-8.5%, -0.7%)	-1.2%<br>(-2.3%, 1.4%)

Supplementary Table 10. Estimated number of cumulative CVD deaths and 95% uncertainty intervals for 2021-2030 associated with each modelled Brexit scenario – Sensitivity analyses

Scenario	Coronary heart disease	Stroke	Cardiovascular disease
Sensitivity Analysis A: Taking into account effect of canned, dried, and frozen F&V			
Scenario 1	1,400 (760, 2,830)	2,860 (1,440, 5,590)	4,260 (2,200, 8,420)
Scenario 2	1,970 (1,010, 4,190)	3,980 (1,940, 8,170)	5,950 (2,940, 12,360)
Scenario 3	4,230 (2,370, 8,070)	8,760 (4,630, 16,720)	12,990 (7,000, 24,790)
Scenario 4	1,390 (830, 2,420)	2,920 (1,610, 5,200)	4,310 (2,430, 7,620)
Sensitivity Analysis B: Increasing domestic production by 2% a year*			
Scenario 1	1,300 (690, 2,550)	2,590 (1,310, 5,190)	3,890 (2,000, 7,730)
Scenario 2	1,850 (930, 3,860)	3,650 (1,790, 7,620)	5,500 (2,720, 11,470)
Scenario 3	3,890 (2,190, 7,300)	7,800 (4,090, 14,880)	11,680 (6,280, 22,180)
Scenario 4	1,260 (740, 2,150)	2,590 (1,420, 4,700)	3,860 (2,150, 6,850)

**Increase in domestic supply was assumed for all F&V excluding bananas, citrus fruits, and grapes.*

Supplementary Table 11. Estimated relative change in intake of F&V in the first year (2021) and last year (2030) of the modelling period under each modelled Brexit scenario, assuming that domestic supply of F&V will increase by approximately 2% a year*. Numbers in parentheses indicate 95% uncertainty intervals.

Scenario	Change in intake	
	Fruits	Vegetables
2021		
Scenario 1	-4.4% (-5.2%, -3.8%)	-2.5% (-3.0%, -1.9%)
Scenario 2	-7.0% (-8.4%, -5.9%)	-2.7% (-3.3%, -2.2%)
Scenario 3	-11.4% (-14.2%, -9.5%)	-9.1% (-11.0%, -7.8%)
Scenario 4	-2.8% (-3.3%, -2.3%)	-4.0% (-4.6%, -3.4%)
2030		
Scenario 1	-4.1% (-4.9%, -3.5%)	-2.1% (-2.5%, -1.6%)
Scenario 2	-6.6% (-8.0%, -5.7%)	-2.3% (-2.7%, -1.8%)
Scenario 3	-10.7% (-13.3%, -8.9%)	-7.6% (-9.2%, -6.5%)
Scenario 4	-2.5% (-2.9%, -2.1%)	-3.3% (-3.8%, -2.8%)

**Increase in domestic supply was assumed for all F&V excluding bananas, citrus fruits, and grapes.*

Supplementary figures

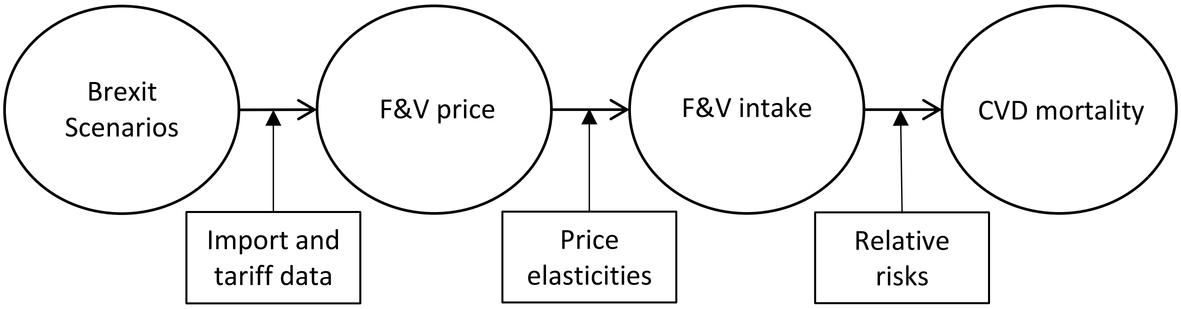

Supplementary Figure 1. Schematic representation of the model and its main inputs.

References

1. WTO. Tariff Analysis Online. Available from: http://tao.wto.org/default.aspx. (accessed February 2018).

2. HM Revenue & Customs. UK Trade Statistics. Available from: https://www.uktradeinfo.com/. (accessed February 2018).

3. Department for Environment Food & Rural Affairs (DEFRA). Horticulture Statistics 2016. . 2017. Available from: https://www.gov.uk/government/collections/horticultural-statistics. (accessed February 2017).

4. World Bank World Integrated Trade Solution (WITS). Available from: https://wits.worldbank.org/default.aspx. (accessed April 2018).

5. Guzman Castillo M, Gillespie DOS, Allen K, et al. Future Declines of Coronary Heart Disease Mortality in England and Wales Could Counter the Burden of Population Ageing. *PLoS One*. 2014;9(6):e99482.

6. Benjamin EJ, Virani SS, Callaway CW, et al. Heart Disease and Stroke Statistics—2018 Update: A Report From the American Heart Association. *Circulation*. 2018.

7. Brownrigg JRW, Hughes CO, Burleigh D, et al. Microvascular disease and risk of cardiovascular events among individuals with type 2 diabetes: a population-level cohort study. *The Lancet Diabetes & Endocrinology*. 2016;4(7):588-97.

8. Abreu MD. Preferential rules of origin in regional trade agreements. World Trade Organization, Economic Research and Statistics Division; 2013.

9. Cadot O, Gourdon J. Non-tariff measures, preferential trade agreements, and prices: new evidence. *Rev World Econ*. 2016;152(2):227-49.

10. Bajekal M, Scholes S, Love H, et al. Analysing Recent Socioeconomic Trends in Coronary Heart Disease Mortality in England, 2000-2007: A Population Modelling Study. *PLoS Med*. 2012;9(6):e1001237.

11. Office for National Statistics, Department for Environment Food and Rural Affairs. Living Costs and Food Survey, 2016-2017. UK Data Service; 2018

12. Micha R, Peñalvo JL, Cudhea F, et al. Association between dietary factors and mortality from heart disease, stroke, and type 2 diabetes in the united states. *JAMA*. 2017;317(9):912-24.

13. Tiffin R, Balcombe K, Salois M, et al. Estimating Food and Drink Elasticities. 2011. Available from: https://www.gov.uk/government/uploads/system/uploads/attachment_data/file/137726/defra-stats-foodfarm-food-price-elasticities-120208.pdf. (accessed February 2017).

14. NatCen Social Research, MRC Elsie Widdowson Laboratory, University College London. Medical School. National Diet and Nutrition Survey Years 1-8, 2008/09-2015/16. 9th Edition ed: UK Data Service; 2018

15. Barendregt JJ. The effect size in uncertainty analysis. *Value Health*. 2010;13(4):388-91.
