# Supplementary material for: Impacts of Brexit on fruit and vegetable intake and cardiovascular disease in England: a modelling study
Source: BMJ Open. 2019 Jan 28;9(1):e026966. doi: 10.1136/bmjopen-2018-026966 (PMC6352794; doi:10.1136/bmjopen-2018-026966)
Supplement: Author's manuscript [file bmjopen-2018-026966.draft_revisions.pdf]

BMJ Open is committed to open peer review. As part of this commitment we make the peer review history of every article we publish publicly available.

When an article is published we post the peer reviewers' comments and the authors' responses online. We also post the versions of the paper that were used during peer review. These are the versions that the peer review comments apply to.

The versions of the paper that follow are the versions that were submitted during the peer review process. They are not the versions of record or the final published versions. They should not be cited or distributed as the published version of this manuscript.

BMJ Open is an open access journal and the full, final, typeset and author-corrected version of record of the manuscript is available on our site with no access controls, subscription charges or pay-per-view fees (http://bmjopen.bmj.com).

If you have any questions on BMJ Open's open peer review process please email info.bmjopen@bmj.com

BMJ Open

Impacts of Brexit on fruit and vegetable intake and cardiovascular disease in England: a modelling study

Journal:	*BMJ Open*
Manuscript ID	bmjopen-2018-026966
Article Type:	Research
Date Submitted by the Author:	28-Sep-2018

Complete List of Authors:	Seferidi, Paraskevi; Imperial College London School of Public Health, Public Health Policy Evaluation Unit<br>Laverty, Anthony; Imperial College London School of Public Health, Public Health Policy Evaluation Unit<br>Pearson-Stuttard, Jonny; Imperial College London School of Public Health<br>Bandosz, Piotr; University of Liverpool, Department of Public Health & Policy<br>Collins, Brendan; University of Liverpool, Department of Public Health and Policy<br>Castillo, Maria; University of Liverpool, Public Health and Policy<br>Capewell, Simon; University of Liverpool, Department of Public Health<br>O'Flaherty, M; University of Liverpool, Department of Public Health<br>Millett, Christopher; Imperial College London School of Public Health, Public Health Policy Evaluation Unit

Keywords:	Brexit, fruits and vegetables, cardiovascular disease, trade policy, diet

SCHOLARONE™ Manuscripts

only

IMPACTS OF BREXIT ON FRUIT AND VEGETABLE INTAKE AND CARDIOVASCULAR DISEASE IN ENGLAND: A MODELLING STUDY

Paraskevi Seferidi[1], Anthony A Laverty[1], Jonathan Pearson-Stuttard[1,2], Piotr Bandosz[2,3], Brendan Collins[2], Maria Guzman-Castillo[2], Simon Capewell[2], Martin O'Flaherty[2], Christopher Millett[1]

[1]Public Health Policy Evaluation Unit, School of Public Health, Imperial College London, London, UK

[2]Department of Public Health and Policy, University of Liverpool, Liverpool, UK; [3]Department of Preventive Medicine and Education, Medical University of Gdańsk, Gdańsk, Poland

Correspondence to: P Seferidi, paraskevi.seferidi14@imperial.ac.uk, Public Health Policy Evaluation Unit, School of Public Health, Imperial College London, Reynolds Building, St Dunstan's Road, W6 8RP, London, UK

Word count: 3,265

For peer review only

For peer review only - http://bmjopen.bmj.com/site/about/guidelines.xhtml

ABSTRACT

Objectives

To estimate the potential impacts of different Brexit trade policy scenarios on the price and intake of fruits and vegetables (F&V) and consequent cardiovascular disease (CVD) deaths in England between 2021 and 2030.

Design

Economic and epidemiological modelling study with probabilistic sensitivity analysis.

Setting

The model combined publicly available data on F&V trade, published estimates of UK-specific price elasticities, national survey data on F&V intake, estimates on the relationship between F&V intake and CVD from published meta-analyses, and CVD mortality projections for 2021-2030.

Participants

English adults aged 25 years and older.

Interventions

We modelled four potential post-Brexit trade scenarios: (1) Free trading agreement with the EU and maintaining half of non-EU free trade partners; (2) Free trading agreement with the EU but no trade deal with any non-EU countries; (3) No deal Brexit; (4) Liberalised trade regime which eliminates all import tariffs.

Outcome measures

Cumulative coronary heart disease and stroke deaths attributed to the different Brexit scenarios modelled between 2021 and 2030.

Results

Under all Brexit scenarios modelled, prices of F&V would increase, especially for those highly dependent on imports. This would decrease intake of F&V between 2.5% (95% Uncertainty Interval: 1.9% to 3.1%) and 11.4% (9.5% to 14.2%) under the different scenarios. Our model suggests that a no deal Brexit scenario would be the most harmful, generating approximately 12,400 (6,690 to 23,390) extra CVD deaths between 2021 and 2030, whereas establishing a free trading agreement with the EU would have a lower impact on mortality, contributing approximately 5,740 (2,860 to 11,910) extra CVD deaths.

For peer review only

For peer review only - http://bmjopen.bmj.com/site/about/guidelines.xhtml

Conclusions

Trade policy under all modelled Brexit scenarios could increase price and decrease intake of F&V, generating substantial additional CVD mortality in England. The UK government should consider the population health implications of Brexit trade policy options, including changes to food systems.

ARTICLE SUMMARY

Strengths and limitations of this study

- This study uses nationally representative dietary intake data, robust mortality projections and both own- and cross-price effect estimates to evaluate the impact of different Brexit scenarios on the price and intake of disaggregated fruit and vegetable subgroups and associated CVD outcomes.
- Fruit and vegetable imports were approximated using the latest available data, assuming import shares will not change after Brexit.
- Trade transaction costs due to technical regulations are based on published estimates that might also reflect costs attributed to demand changes thus overestimating real transaction costs.
- Response to price increases of fruits and vegetables were estimated using historical purchase data and might not reflect consumer response to price change post-Brexit.
- The scenarios modelled are not exhaustive and more trade policy options might arise as Brexit negotiations continue.

For peer review only

For peer review only - http://bmjopen.bmj.com/site/about/guidelines.xhtml

INTRODUCTION

Trade policy exerts a powerful influence on risk factors for non-communicable disease and thus for population health.[1] One prominent mechanism is the effect of trade on food environments, through changes in the availability and affordability of food commodities.[2] Evidence suggests that entering new trading blocs and opening domestic markets to international trade has been associated with increased imports, sales, and intake of highly processed foods.[3-6] However, there is a lack of evidence on the effect of trade on healthy food commodities, while no previous studies have investigated the potential impacts of exiting a large trading bloc.

Brexit – the impending exit of the United Kingdom (UK) from the European Union (EU) – will have profound implications across a range of sectors. One of the main changes will involve the introduction of a new trade regime that will redefine the trade relationships between the UK and the EU and non-EU countries. This is likely to affect food environments in the UK, given its high dependency on imports to meet its dietary needs.[7] This is particularly the case for fruits and vegetables (F&V). For example, in 2017, 84% of fruits and 43% of vegetables in the UK were imported.[8] Thus, changes to the UK trade regime are likely to affect prices of F&V by increasing costs of trade. With price being one of the main determinants of consumer behaviour in the UK,[9] Brexit could affect F&V intake with significant implications for UK diets.

Low F&V intake is a major risk factor of many non-communicable diseases, including cardiovascular disease (CVD). Approximately 1.3 million CVD deaths could have been prevented globally in 2013 if intake of F&V was higher than 500 grams/day (g/d).[10] The UK performs poorly in terms of F&V intake; only 27% of adults aged 19-64 years and 35% of those above 65 years achieve daily recommended intakes.[11] Thus, Brexit trade scenarios that change the price and availability of F&V are likely to have substantial impacts to CVD outcomes in the UK. This analysis aimed to quantify the potential impacts of F&V price changes due to Brexit on CVD deaths in England between 2021 and 2030.

METHODS

We extended the previously validated IMPACT Food Policy model[12-14] to estimate the effect of different Brexit scenarios on the price and intake of F&V in England. We then modelled consequent impacts on cumulative coronary heart disease (CHD) and stroke mortality in English adults aged 25 and above, stratified by age and sex, from 2021-2030.

For peer review only - http://bmjopen.bmj.com/site/about/guidelines.xhtml

Data sources

We used import and tariff data from the World Trade Organization (WTO)[15] and the HM Revenues and Customs (HMRC)[16] to estimate the effect of different Brexit scenarios on price of F&V. We describe these data in more detail in Appendix A.

The effect of price changes on intake of F&V products was estimated using published UK-specific price elasticities.[17] Price elasticities measure the change in demand of a good as a response to a change in its own price (own-price elasticity) or the price of another good (cross-price elasticity). Price elasticities for five fruit (Apples and Pears; Bananas; Citrus fruit; Grapes; Other fruits) and seven vegetable (Brassica, such as cabbages, cauliflower, and broccoli; Legumes, such as beans and peas; Lettuce; Onions; Other vegetables; Root vegetables; Tomatoes) subgroups were available. We used conditional uncompensated own and cross-price elasticities to take into account the differential effect of Brexit on the prices of different F&V. Conditional price elasticities, usually recommended at this level of product disaggregation, assume that food expenditure available to all other products remains constant, and uncompensated price elasticities assume that consumer income is held constant.[17]

We estimated total F&V intake in English adults by sex and age (25-44, 45-74, 75+) using data from the National Diet and Nutrition Survey Rolling Programme (2008/09-2015/16).[18] This is a nationally representative survey of UK adults and children that uses a four-day food diary to evaluate dietary intake. As the effect of the different Brexit scenarios varies across different F&V products, we used household purchase data for England from the Family Food Module of the Living Costs and Food Survey 2016/17[19] to approximate intake of F&V subgroups. The survey uses a two-week diary to identify purchases and expenditures of foods in UK households. We adjusted the overall F&V intake using the ratio of purchases of F&V subgroups with overall F&V purchases. We assumed a constant ratio across all sex and age groups.

Changes in F&V intake were translated into mortality changes using relative risk estimates from meta-analyses of longitudinal studies[20] that provide age-specific associations between fruit intake, vegetable intake and CHD, ischemic stroke, and haemorrhagic stroke. We assumed a linear relationship between risk and mortality of the outcomes.

Finally, this model employed sex and age (10-year groups from 25-34 until 85+) specific CVD mortality projections between 2021 and 2030 which were estimated using a Bayesian Age-Period-Cohort model and historic CVD mortality and population data from the Office for National Statistics. The methodology is described in more detail in Appendix B.

The data and data sources used in this model are summarised in Table 1.

For peer review only - http://bmjopen.bmj.com/site/about/guidelines.xhtml

Table 1. Model inputs and data sources

Data	Data source
Import and tariff data	World Trade Organisation[15] and HM Revenues and Customs[16]
Supply data (domestic production, imports, and exports)	Department for Environment, Food and Rural Affairs[21]
Price elasticities (mean of annual elasticity estimates between 2000-2009)	Tiffin, 2011[17]
Means of F&V intake by age and sex	National Diet and Nutrition Survey Rolling Programme Years 1-8 (2008/09-2015/16)[18]
F&V subgroups gradient (ratio to overall F&V intake)	Purchase data from the Living Costs and Food Survey 2016/17[19]
Relative Risk for CHD/ ischemic stroke/ haemorrhagic stroke by serving of fruit/vegetable intake by age	Micha, 2017[20]
CHD and stroke mortality projections for England by age and sex (2021-2030)	Own estimations using historic mortality data and population projections from the Office for National Statistics
Ischemic to Haemorrhagic stroke ratio, estimated using number of deaths by ICD-code, age, and sex in 2016	Office for National Statistics

Modelled scenarios

The UK, as an EU Member State, is part of the EU Single Market and Customs Union. As a result, trade between the UK and the EU is tariff-free and frictionless with no tariff or non-tariff trade barriers in place.[22] Trade between the UK and non-EU countries is also regulated by the EU, with common preferential trade agreements and external tariffs across all EU Member States. At an international level, trade is regulated by the WTO which is used as a platform for its members to negotiate and ratify trade agreements.

Following the referendum in June 2016, the UK government has announced its intentions to leave the EU Single Market and Customs Union and negotiate a new trading relationship with the EU and third countries.[23] The current intention from both the UK and the EU is that this will happen two years after Brexit, following a transition period that will maintain the trade status quo while allowing the UK to negotiate a future permanent trading position.

For this analysis, we modelled four possible post-Brexit trade scenarios commencing in 2021 when the transition period will end:

1. Free trading agreement with the EU and third countries (S1).

For peer review only - http://bmjopen.bmj.com/site/about/guidelines.xhtml

In this scenario, the UK establishes a zero-tariff free trading agreement with the EU while it also maintains half of its non-EU tariff-free F&V importers.

2. Free trading agreement with the EU (S2).

   Under this scenario, the UK establishes a zero-tariff free trading agreement with the EU but loses all preferential access to markets of non-EU F&V importers.

3. No deal Brexit (S3).

   Under a no deal scenario the UK falls back into a WTO default position, having no preferential arrangements with the EU or non-EU importers.

4. Liberalised trade regime (S4).

   Under a liberalised trade regime, the UK trades under WTO regulations with no specific trade arrangements in place, but eliminates all its F&V import tariffs.

Under each scenario, we assumed two main drivers of F&V import price change. First, we expect an increase in transaction costs when trading with the EU due to a rise in post-Brexit border controls. We assumed a 5% increase in transaction costs due to rules of origin checks[24] and a 4.5% increase due to checks for technical regulations.[25] We describe these estimates and its sources in detail in Appendix C. Second, we assumed changes in F&V import tariffs[15] that vary under the different Brexit scenarios. Calculations for each scenario are described in Appendix C. The estimated change in price of F&V imports was translated into change in wholesale F&V price using an estimate of imports as a percentage of overall F&V supply in the UK, obtained from the Department for Environment, Food & Rural Affairs (DEFRA)[21] (see Appendix A for more details).

The IMPACT Food Policy model

The IMPACT Food Policy model has been previously used to estimate the effect of different policies on dietary intake and CVD in the UK[12] and elsewhere.[26] We extended the model to estimate the effect of different Brexit scenarios on prices of F&V subgroups as described above. We then translated changes in prices into changes in intake taking into account potential substitution between subgroups. We allowed the price of each F&V subgroup to change one by one until final equilibrium was reached, i.e. when prices of all F&V subgroups had changed. The intake change was the difference between intake at final equilibrium and baseline. Finally, the model translated changes in intake into changes in mortality using appropriate relative risks. We assumed common relative risks for all fruit and all vegetable subgroups. The risk reduction effect of overall F&V was estimated using a cumulative risk-reduction approach.[27] Changes in mortality were expressed as number of deaths attributed to each Brexit scenario. CVD mortality was estimated as the sum of CHD, ischemic stroke, and haemorrhagic stroke. Calculations are described in more detail in Appendix D.

For peer review only - http://bmjopen.bmj.com/site/about/guidelines.xhtml

Uncertainty and sensitivity analyses

We performed a probabilistic sensitivity analysis to take into account uncertainty of model inputs. We employed Monte Carlo simulations to repeatedly draw random values of the inputs from their statistical distributions and run the model across multiple iterations. The estimates and their respective statistical distributions are presented in Supplementary Table 1. The median and 2.5 and 97.5 percentiles of 1,000 iterations were used to produce estimates and their 95% uncertainty intervals (95% UI).

As a response to an increase in the price of F&V, consumers may shift to other F&V alternatives. We performed an additional sensitivity analysis to take into account possible substitution between fresh F&V and canned, dried, and frozen F&V. The effect of the different Brexit scenarios on price of canned, dried, and frozen F&V was estimated following the same methodology as the main analysis. Then, cross-price elasticities were used to identify the effect of change in the price of canned, dried, and frozen F&V on fresh F&V. Finally, the effect of change in intake of fresh F&V on CVD mortality was calculated. Given the absence of evidence, we assumed that canned, dried, and frozen F&V were not associated with CVD outcomes. Details about data sources and calculations are presented in Appendix E.

Finally, we performed a second additional sensitivity analysis that allows domestic production of F&V to increase as a response to the increase in price of imported F&V. We assumed a 2% increase per year for all F&V subgroups except bananas, citrus fruits, and grapes which have currently no domestic production in the UK.

Patient and public involvement

There was no patient and public involvement in this study.

RESULTS

F&V prices

The estimated effect of Brexit scenarios on price of F&V subgroups are presented in Figure 1. Price effects varied among different F&V subgroups, depending on the origin of imports, the applied tariffs, and the percentage of supply that is imported.

The no deal Brexit scenario (S3) would affect prices of F&V the most, especially for F&V that are highly dependent on imports. For example, the model suggested that under a no deal Brexit scenario, prices would increase by 16.9% (95% UI: 16.6% to 17.1%) for bananas, 14.3% (7.6% to 27.2%) for citrus fruit, and 14.9% (13.2% to 16.6%) for tomatoes. In contrast, prices of root vegetables, whose imports contribute only 5% to their total supply, would increase between 0.3%

For peer review only - http://bmjopen.bmj.com/site/about/guidelines.xhtml

(0.1% to 0.5%) and 1.1% (0.6% to 1.7%) under different Brexit scenarios. A liberalised trade regime (S4) would mostly affect prices of F&V that are primarily imported from the EU, like citrus fruits and tomatoes with an estimated 4.2% (3.0% to 5.6%) and 6.0% (4.4% to 7.7%) increase in price respectively. More detailed price effects are presented in Supplementary Table 2.

F&V intake

We estimated that mean intake of F&V at baseline was 111 g/d and 190 g/d respectively (Supplementary Table 3). The most consumed fruits were bananas (26% of fruit intake) and apples and pears (19% of fruit intake), while root vegetables were the most consumed vegetables (17% of vegetable intake) (Supplementary table 4).

We estimated that intake of F&V would reduce under all Brexit scenarios (Table 2). The no deal Brexit scenario (S3) would have the largest effect, reducing intake of fruits by approximately 11.4% (9.5% to 14.2%) and vegetables by approximately 9.1% (7.8% to 11.0%). If a free trading agreement with the EU is established (S2), vegetable intake would reduce by 2.7% (2.2% to 3.3%), whereas intake of fruits would reduce by 7.0% (5.9% to 8.4%). Intake effects on F&V subgroups are presented in Supplementary Table 5.

Table 2. Estimated relative change in intake of F&V under each modelled Brexit scenario.

Scenario	Change in intake (95% UI)	
	Fruits	Vegetables
Scenario 1	-4.4% (-5.2% to -3.8%)	-2.5% (-3.1% to -1.9%)
Scenario 2	-7.0% (-8.4% to -5.9%)	-2.7% (-3.3% to -2.2%)
Scenario 3	-11.4% (-14.2% to -9.5%)	-9.1% (-11.0% to -7.8%)
Scenario 4	-2.8% (-3.3% to -2.3%)	-4.0% (-4.6% to -3.4%)

Scenario S1: Free trading agreement with the EU and third countries; Scenario S2: Free trading agreement with the EU; Scenario S3: No deal Brexit; Scenario S4: Liberalised trade regime

CVD mortality

The number of deaths attributable to each Brexit scenario are presented in Table 3 and Figure 2. The no deal Brexit scenario (S3) would be the most impactful, contributing approximately 12,400 (6,690 to 23,390) extra CVD deaths or a 1.7% increase in CVD mortality over the modelling period of 2021-2030. Establishing a free trading agreement with the EU (S2) would have lower impacts on mortality contributing approximately 5,740 (2,860 to 11,910) extra CVD deaths or a 0.8% increase in CVD mortality over the ten-year period. Free trading agreements with both the EU and half of the UK's F&V importers (S1) would have more attenuated impacts, contributing approximately 4,110 (2,130 to 8,100) extra CVD deaths or a 0.6% increase in CVD mortality. Even under a liberalised regime that eliminates all import tariffs (S4), Brexit would contribute approximately 4,160 (2,330 to 7,380) extra

For peer review only - http://bmjopen.bmj.com/site/about/guidelines.xhtml

CVD deaths or a 0.6% increase in CVD mortality between 2021 and 2030 due to an increase in transaction costs of EU imports.

Table 3. Estimated number of cumulative CVD deaths and 95% UI for 2021-2030 associated with each modelled Brexit scenario

Scenario	Coronary heart disease	Stroke	Cardiovascular disease
Scenario 1	1,360 (730 to 2,670)	2,740 (1,400 to 5,430)	4,110 (2,130 to 8,100)
Scenario 2	1,930 (980 to 3,990)	3,810 (1,880 to 7,910)	5,740 (2,860 to 11,910)
Scenario 3	4,110 (2,330 to 7,660)	8,290 (4,360 to 15,730)	12,400 (6,690 to 23,390)
Scenario 4	1,360 (790 to 2,300)	2,810 (1,540 to 5,080)	4,160 (2,330 to 7,380)

Scenario S1: Free trading agreement with the EU and third countries; Scenario S2: Free trading agreement with the EU; Scenario S3: No deal Brexit; Scenario S4: Liberalised trade regime

Sensitivity Analyses

Our model suggested that prices of canned, dried, and frozen F&V would increase by approximately 10.3% (5.7% to 20.0%) for fruits and 6.8% (3.9% to 16.7%) for vegetables under a no deal Brexit scenario (S3), as shown in Supplementary Table 7. Incorporating cross-price elasticities between fresh and canned, dried, and frozen F&V in the model only slightly increased the effect of Brexit scenarios on intake of fresh F&V by up to 0.5% (Supplementary Table 8). This would result to approximately 12,990 (7,000 to 24,790) extra CVD deaths between 2021 and 2030 under a no deal Brexit scenario (S3) (Supplementary Table 9).

Our model that examined potential increases in the domestic production of some F&V did not substantially change our findings. The estimated effect on intake was 0.7% lower for fruits and 1.5% for vegetables in the last year of the modelling period compared to the first year, under a no deal Brexit scenario (S3) (Supplementary Table 10). Over the study period, this would contribute to between 210 and 720 fewer CVD deaths under the different scenarios compared to the main analysis for which domestic production was assumed to be stable (Supplementary Table 9).

DISCUSSION

This is the first study to quantify the potential impacts of post-Brexit trade scenarios on F&V prices, intake, and consequent CVD outcomes. Estimates from all modelled scenarios suggested that F&V prices would increase, hence reducing intake and contributing to additional CVD mortality. A no deal Brexit scenario could be the most harmful, increasing CHD and stroke deaths by approximately 0.9% (4,110 deaths) and 2.9% (8,290 deaths) respectively between 2021 and 2030. The least disruptive scenario modelled, which assumes a free trading agreement with the EU and half of non-EU F&V importers, could increase CHD and stroke deaths by approximately 0.3% (1,360 deaths) and 1.0% (2,740 deaths) respectively.

For peer review only - http://bmjopen.bmj.com/site/about/guidelines.xhtml

Our findings are consistent with previous preliminary analyses which suggest likely increases in F&V prices post-Brexit. A report from the House of Lords notes the vulnerability of F&V to price changes post-Brexit.[28] An empirical estimation showed that the cost of consuming five F&V per day as per governmental guidelines will increase by £2.20 a week for a household of four under a no deal Brexit scenario, after considering price inflation and an increase in import tariffs.[29] Analysis using a partial equilibrium model, which uses supply and demand curves to examine the effect of policy actions on single markets, has also shown that a no deal Brexit might increase prices of fruits by 3.1% and vegetables by 4.0% although disaggregated effects on F&V subgroups were not further investigated.[30] These analyses did not compare a no deal Brexit scenario with other potential post-Brexit trade options. Furthermore, they did not account for the potential effect of non-tariff trade barriers which are likely to be an important driver of price change post-Brexit.

This study has several strengths. It estimates the effect of different Brexit scenarios on the price of disaggregated F&V subgroups, taking into account both own- and cross-price effects to estimate changes in F&V intake. Sensitivity analyses also incorporated the additional effects of substitution with canned, dried, and frozen F&V and increasing domestic production of F&V. This study uses nationally representative data on F&V intake and purchases. It also employs CVD mortality projections using a Bayesian Age-Period-Cohort model which has been shown to produce more robust predictions compared with conventional methods.[31]

However, it also has some limitations. We used the most recent import data from 2015 to approximate F&V imports post-Brexit, assuming that F&V import shares will not change. There might be some discrepancies in the definition of F&V subgroups across different datasets. We explicitly describe each definition in Appendix A. Trade transaction costs due to technical regulations are based on published estimates derived using non-tariff measure data from 65 countries.[25] These estimates might overestimate the real costs as they might also reflect a price effect attributed to an increase in demand after complying with quality improving technical regulations in some countries. We used robust estimates of UK specific price elasticities.[17] However, these elasticities were estimated using historical purchase data (2000-2009) and might not reflect the consumer response to price change after a potential wider economic shock of Brexit. Finally, we modelled four plausible post-Brexit trade scenarios reflecting governmental and expert consensus.[32] However, ongoing negotiations within the British government and the Conservative party[33] and between the British government and the EU[34] means that the political space around Brexit is continuously changing and more trade policy options might arise.

F&V intake in the UK is suboptimal with less than half of the population consuming daily recommended levels.[11] Our analysis suggests that all modelled Brexit trade policy scenarios could

For peer review only - http://bmjopen.bmj.com/site/about/guidelines.xhtml

increase price and reduce intake of F&V. The UK government needs to develop a comprehensive policy response against this potential detrimental impact by aligning domestic nutrition and agricultural policies and future trade policy with public health aims. At the same time, the wider implications of Brexit to the UK food system should also be considered. For example, approximately 80,000 seasonal workers are employed in the UK every year to harvest F&V, with the majority of them recruited from EU countries.[35] Abolishment of the free movement of labour guaranteed by EU regulations is likely to reduce available workforce for the horticulture sector with potential implications to prices of domestically produced F&V. Brexit may also affect food quality and safety in the UK by potentially relaxing risk prevention requirements, like the precautionary principle for food which is currently eliminated from the EU (Withdrawal) Act 2018,[36] in order to increase competitiveness in international markets. Moreover, the wider effects of Brexit on the UK economy could affect the whole food supply chain, including packaging, distribution, and retail of both fresh and processed foods. Future work should monitor and evaluate the population health implications of these potentially important changes in the UK food system.

In summary, post-Brexit trade policy could increase price and decrease intake of F&V, thus increasing CVD mortality in England. The UK government should therefore carefully consider the population health implications of Brexit during upcoming negotiations and post-Brexit planning, particularly adverse changes to food systems.

Contributor information: PS, AL, JPS, MOF, CM conceived the study. PS and PB analysed the data with inputs from all authors. PS drafted the paper with inputs from all authors. All authors made a substantial contribution to the data interpretation and critical review of the submitted manuscript.

Funding: PS, AL and CM are funded by the National Institute for Health Research (NIHR) via a Research Professorship Award to CM (grant number: RP 2014-04-032). NIHR had no role in the design, analysis or writing of this article. The Public Health Policy Evaluation Unit is grateful for the support of the NIHR School of Public Health Research.

Competing interests: None

Data sharing: Model input data are available on request from the corresponding author.

For peer review only - http://bmjopen.bmj.com/site/about/guidelines.xhtml

FIGURES

Figure 1. Estimated relative change in price of fruit and vegetable subgroups under each modelled Brexit scenario. Error bars indicate 95% uncertainty intervals.

Scenario S1: Free trading agreement with the EU and third countries; Scenario S2: Free trading agreement with the EU; Scenario S3: No deal Brexit; Scenario S4: Liberalised trade regime

Figure 2. Estimated number of cumulative CVD deaths for 2021-2030 associated with each modelled Brexit scenario, by sex.

Scenario S1: Free trading agreement with the EU and third countries; Scenario S2: Free trading agreement with the EU; Scenario S3: No deal Brexit; Scenario S4: Liberalised trade regime

For peer review only

For peer review only - http://bmjopen.bmj.com/site/about/guidelines.xhtml

REFERENCES

1. Schram A, Ruckert A, VanDuzer JA, et al. A conceptual framework for investigating the impacts of international trade and investment agreements on noncommunicable disease risk factors. *Health Policy Plan*. 2018;33(1):123-36.

2. Friel S, Hattersley L, Snowdon W, et al. Monitoring the impacts of trade agreements on food environments. *Obes Rev*. 2013;14 Suppl 1:120-34.

3. Stuckler D, McKee M, Ebrahim S, et al. Manufacturing Epidemics: The Role of Global Producers in Increased Consumption of Unhealthy Commodities Including Processed Foods, Alcohol, and Tobacco. *PLoS Med*. 2012;9(6):e1001235.

4. Baker P, Friel S. Food systems transformations, ultra-processed food markets and the nutrition transition in Asia. *Global Health*. 2016;12(1):80.

5. Thow AM, Hawkes C. The implications of trade liberalization for diet and health: a case study from Central America. *Global Health*. 2009;5(1):5.

6. Barlow P, McKee M, Basu S, et al. Impact of the North American Free Trade Agreement on high-fructose corn syrup supply in Canada: a natural experiment using synthetic control methods. *Cmaj*. 2017;189(26):E881-e7.

7. Department for Environment Food & Rural Affairs (DEFRA). Food statistics pocketbook 2017. 2017. Available from: https://www.gov.uk/government/collections/food-statistics-pocketbook. (accessed July 2018).

8. Department for Environment Food & Rural Affairs (DEFRA). Agriculture in the United Kingdom 2017.; 2018.

9. Farrow L, Georgieva I. Trade-offs in future food systems – consumer perspectives. A GFS Food Futures panel project. 2016.

10. Aune D, Giovannucci E, Boffetta P, et al. Fruit and vegetable intake and the risk of cardiovascular disease, total cancer and all-cause mortality—a systematic review and dose-response meta-analysis of prospective studies. *Int J Epidemiol*. 2017;46(3):1029-56.

11. Caireen Roberts, Toni Steer, Natalie Maplethorpe, et al. National Diet and Nutrition Survey Results from Years 7 and 8 (combined) of the Rolling Programme (2014/2015 to 2015/2016). Public Health England; 2018.

12. Allen K, Pearson-Stuttard J, Hooton W, et al. Potential of trans fats policies to reduce socioeconomic inequalities in mortality from coronary heart disease in England: cost effectiveness modelling study. *BMJ*. 2015;351:h4583.

13. Pearson-Stuttard J, Critchley J, Capewell S, et al. Quantifying the Socio-Economic Benefits of Reducing Industrial Dietary Trans Fats: Modelling Study. *PLoS One*. 2015;10(8):e0132524.

14. Collins M, Mason H, O'Flaherty M, et al. An Economic Evaluation of Salt Reduction Policies to Reduce Coronary Heart Disease in England: A Policy Modeling Study. *Value in Health*. 2014;17(5):517-24.

15. WTO. Tariff Analysis Online. Available from: http://tao.wto.org/default.aspx. (accessed February 2018).

16. HM Revenue & Customs. UK Trade Statistics. Available from: https://www.uktradeinfo.com/. (accessed February 2018).

17. Tiffin R, Balcombe K, Salois M, et al. Estimating Food and Drink Elasticities. 2011. Available from: https://www.gov.uk/government/uploads/system/uploads/attachment_data/file/137726/defra-stats-foodfarm-food-price-elasticities-120208.pdf. (accessed February 2017).

18. NatCen Social Research, MRC Elsie Widdowson Laboratory, University College London. Medical School. National Diet and Nutrition Survey Years 1-8, 2008/09-2015/16. 9th Edition ed: UK Data Service; 2018

For peer review only - http://bmjopen.bmj.com/site/about/guidelines.xhtml

19. Office for National Statistics, Department for Environment Food and Rural Affairs. Living Costs and Food Survey, 2016-2017. UK Data Service; 2018
20. Micha R, Peñalvo JL, Cudhea F, et al. Association between dietary factors and mortality from heart disease, stroke, and type 2 diabetes in the united states. *JAMA*. 2017;317(9):912-24.
21. Department for Environment Food & Rural Affairs (DEFRA). Horticulture Statistics 2016. . 2017. Available from: https://www.gov.uk/government/collections/horticultural-statistics. (accessed February 2017).
22. EU Customs Union. Available from: http://trade.ec.europa.eu/tradehelp/eu-customs-union. (accessed July 2018).
23. HM Government. The United Kingdom's exit from and new partnership with the European Union. 2017.
24. Abreu MD. Preferential rules of origin in regional trade agreements. World Trade Organization, Economic Research and Statistics Division; 2013.
25. Cadot O, Gourdon J. Non-tariff measures, preferential trade agreements, and prices: new evidence. *Rev World Econ*. 2016;152(2):227-49.
26. Pearson-Stuttard J, Bandosz P, Rehm CD, et al. Reducing US cardiovascular disease burden and disparities through national and targeted dietary policies: A modelling study. *PLoS Med*. 2017;14(6):e1002311.
27. Bajekal M, Scholes S, Love H, et al. Analysing Recent Socioeconomic Trends in Coronary Heart Disease Mortality in England, 2000-2007: A Population Modelling Study. *PLoS Med*. 2012;9(6):e1001237.
28. HOUSE OF LORDS European Union Committee. Brexit: food prices and availability. 2018.
29. The Food Foundation. FARMING FOR 5-A-DAY: Brexit Bounty or Dietary Disaster? ; 2017.
30. Clarke S, Serwicka I, Winters LA. Will Brexit Raise the Cost of Living? *National Institute Economic Review*. 2017;242(1):R37-R50.
31. Guzman Castillo M, Gillespie DOS, Allen K, et al. Future Declines of Coronary Heart Disease Mortality in England and Wales Could Counter the Burden of Population Ageing. *PLoS One*. 2014;9(6):e99482.
32. House of Commons International Trade Committee. . UK trade options beyond 2019. 2017.
33. HM Government. The future relationship between the United Kingdom and the European Union. 2018.
34. European Commission - Speech - Statement by Michel Barnier at the press conference following his meeting with Dominic Raab, UK Secretary of State for Exiting the EU [press release]. 2018.
35. NFU Access to a competent and flexible workforce. 2017.
36. European Union (Withdrawal) Act 2018. Available from: https://services.parliament.uk/bills/2017-19/europeanunionwithdrawal.html. (accessed July 2018).

For peer review only - http://bmjopen.bmj.com/site/about/guidelines.xhtml

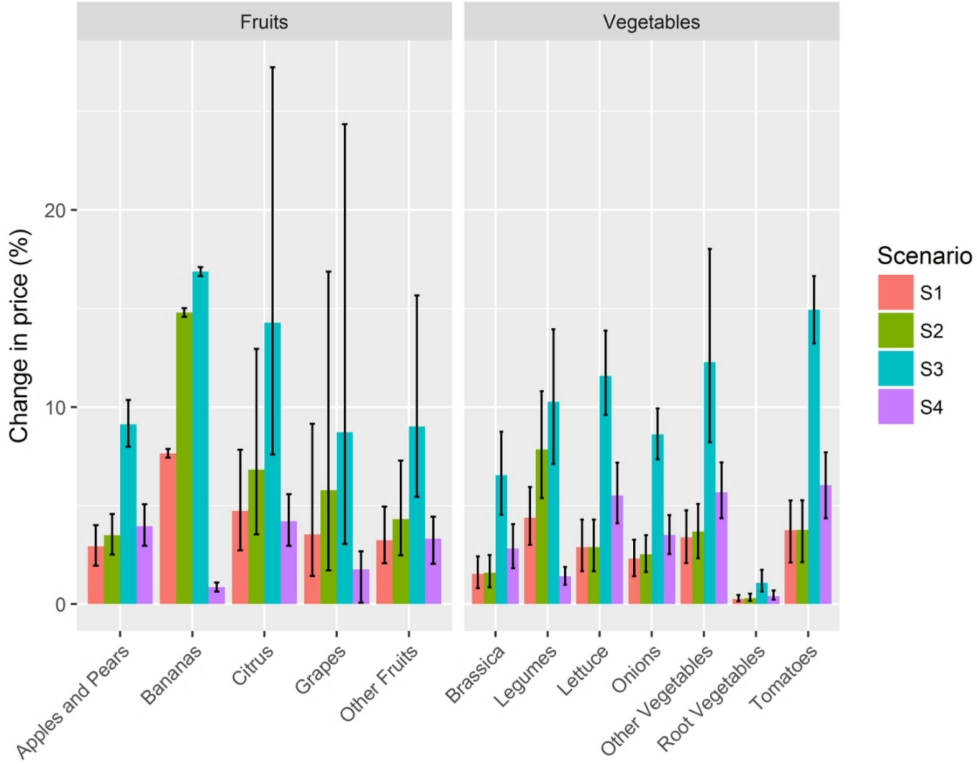

Figure 1. Estimated relative change in price of fruit and vegetable subgroups under each modelled Brexit scenario. Error bars indicate 95% uncertainty intervals.
Scenario S1: Free trading agreement with the EU and third countries; Scenario S2: Free trading agreement with the EU; Scenario S3: No deal Brexit; Scenario S4: Liberalised trade regime

129x105mm (300 x 300 DPI)

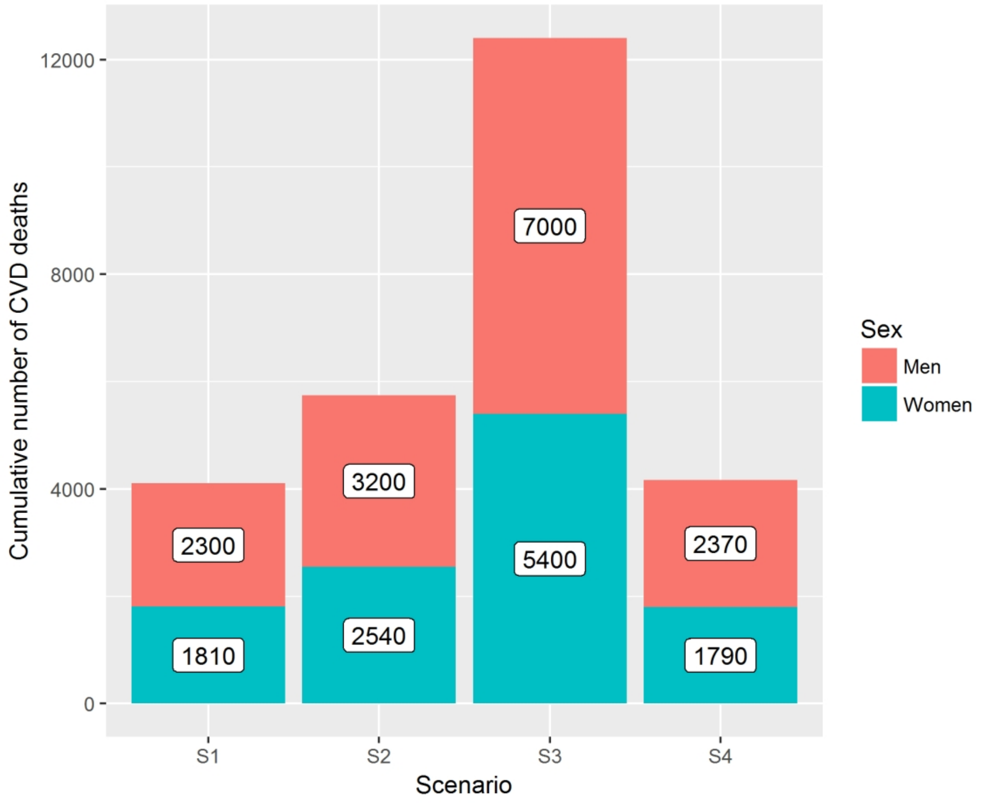

Figure 2. Estimated number of cumulative CVD deaths for 2021-2030 associated with each modelled Brexit scenario, by sex.
Scenario S1: Free trading agreement with the EU and third countries; Scenario S2: Free trading agreement with the EU; Scenario S3: No deal Brexit; Scenario S4: Liberalised trade regime

159x129mm (300 x 300 DPI)

Supplementary Appendix

Appendix A. Trade data inputs

The data inputs used in the policy layer of the model are presented in Table A1. These include fruit and vegetable (F&V) imports to the UK from the EU and from non-EU countries, either dutiable or under a duty-free arrangement, estimates of applied import tariffs, and estimates of imports as a percentage of overall F&V supply. We used a combination of data from the World Trade Organization (WTO),(1) the HM Revenues and Customs (HMRC),(2) and the Department for Environment, Food & Rural Affairs (DEFRA)(3) to obtain these estimates. The methodology used is described in detail below.

F&V were classified into five groups of fruits and seven groups of vegetables, in accordance to the classification applied by Tiffin et al. to estimate the price elasticities used in this model. We also identified two categories of canned, dried, and frozen F&V that were used in the sensitivity analysis. We used the Harmonised System (HS) 2012 classification to assign traded commodities to these groups. The HS has been developed by the World Customs Organization and has international applications, facilitating trade across participating countries. It uses a six-digit code system, arranged in three levels of disaggregation (two-digit, four-digit, and six-digit) to classify commodities. In this analysis, we used the four-digit HS level. The commodities included in each subgroup of F&V are presented in Table A2.

We used the latest available data (2015) from the HMRC(2) to identify imports of F&V (in net mass, kg) to the UK by country of origin for each F&V subgroup. Using information from the WTO, we identified the duty arrangement that is in place between each of the UK's F&V importers and the EU. These included bilateral or multilateral preferential arrangements or, if no agreement was in place, trade under the Most Favoured Nation (MFN) principle. The MFN principle prevents WTO members from discriminating between countries by imposing a common MFN duty to all their trading partners that are not part of a preferential trade agreement. We, then, identified which preferential arrangements allowed for duty-free trade for each F&V subgroup. As different duty rates are applied to the single products that comprise each commodity group (tariff lines), we defined as a duty-free arrangement every arrangement that gave duty-free access to at least 50% of tariff lines in each F&V subgroup.

We used data from the WTO(1) to estimate the mean ad valorem MFN duty applied by the EU and its standard deviation for each F&V subgroup. The WTO provides several averaging methods for applied tariffs in each commodity group. We used the HS sub-heading averaging method which gives equal importance to all HS sub-headings included in each commodity group.(1) The standard

For peer review only - http://bmjopen.bmj.com/site/about/guidelines.xhtml

deviation shows the dispersion of HS sub-heading averages around the overall simple average of the commodity group. This method is not biased by the different levels of disaggregation in the applied tariffs across WTO members, allowing for a uniform comparison across countries. When more than one commodity code was included in our F&V subgroups (i.e. for other and canned, dried, and frozen F&V), we used the same method to average across groups by summing the average tariff of each commodity code and dividing by the total number of subheadings included in the group. The combined standard deviation for these groups was estimated using the following formula:

$$Combined\ SD = \frac{\sqrt{\sum(n*(s^2+(\bar{x}-\bar{X})^2)}}{\sum n}$$

where *n* is the number of subheadings, *s* the standard deviation for each subheading, $\bar{x}$ the average tariff for each subheading, and $\bar{X}$ the average tariff of all subheadings.

Tariffs for all F&V subgroups were ad valorem with the exception of tomatoes which have a specific duty defined in €/100 kg. The World Bank provides estimations of the ad valorem equivalent of specific duties.(4) These varied based on the declared price of tomatoes. We conservatively used the smaller tariff provided, as tomato price is seasonal and fluctuates significantly thought out the year.

Finally, we estimated F&V imports as a percentage of F&V supply. DEFRA provides information on imports, exports, and production of F&V.(3) Total supply was defined as the sum of domestic production and imports minus exports. We estimated imports as a percentage of total supply for years 2006 to 2016. We then used the mean and standard deviation of these estimates for our model. We assigned the F&V products provided by DEFRA into our F&V subgroups as shown in Table A2.

For peer review only

For peer review only - http://bmjopen.bmj.com/site/about/guidelines.xhtml

Table A1. Trade related inputs of the model policy layer.

	Imports from the EU (% of total imports)	Duty-free imports from non-EU countries (% of total imports)	Dutiable imports from non-EU countries (% of total imports)	Ad valorem EU MFN duty (%) Mean (std dev)	Imports (% of supply) Mean (std dev)
Fruits					
Apples and Pears	67.1	21.5	11.4	7.2 (0.0)	71.5 (2.4)
Bananas	10.2	89.2	0.6	16.0 (0.0)	100.0 (0)
Citrus fruit	51.2	43.1	5.7	10.8 (5.5)	100.0 (0)
Grapes	26.2	63.8	10.1	8.4 (6.0)	100.0 (0)
Other Fruits	49.2	38.2	12.6	7.0 (3.4)	87.0 (1.2)
Vegetables					
Brassica	97.0	2.7	0.3	12.0 (0.0)	31.0 (4.6)
Legumes	18.7	78.5	2.8	9.6 (1.6)	92.6 (8.2)
Lettuce	99.8	0.1	0.0	10.4 (0.0)	58.8 (4.1)
Onions	82.4	8.5	9.2	10.0 (0.4)	50.9 (2.5)
Other vegetables	88.5	9.0	2.5	9.8 (3.5)	70.6 (2.1)
Root vegetables	86.2	13.4	0.3	13.3 (0.3)	5.1 (1.3)
Tomatoes	88.6	0.3	11.2	10.8 (0.0)	82.8 (1.2)
Canned, dried, and frozen					
Fruits	61.7	22.6	15.7	8.1 (5.2)	87.0 (1.2)
Vegetables	62.5	7.2	30.3	7.3 (6.8)	70.6 (2.1)

Table A2. Classification of F&V across different data sources.

	Trade and Tariff data (HS 4-digit classification)	Supply data (DEFRA)	Living Costs and Food Survey – household purchases
Fruits			
Apples and Pears	0808 - Apples, pears and quinces, fresh	Total Dessert Apples; Total Culinary Apples; Total Pears	Fresh apples, Fresh pears
Bananas	0803 - Bananas, incl. plantains, fresh or dried	Bananas	Fresh bananas
Citrus fruit	0805 - Citrus fruit, fresh or dried	Citrus	Fresh oranges, Other fresh citrus fruits
Grapes	0806 - Grapes, fresh or dried	Grapes	Fresh grapes

For peer review only - http://bmjopen.bmj.com/site/about/guidelines.xhtml

For peer review only

Other Fruits	0804 - Dates, figs, pineapples, avocados, guavas, mangoes and mangosteens, fresh or dried; 0807 - Melons, incl. watermelons, and papaws "papayas", fresh; 0809 - Apricots, cherries, peaches incl. nectarines, plums and sloes, fresh; 0810 - Fresh strawberries, raspberries, blackberries, back, white or red currants, gooseberries and other edible fruits	Total Plums; Cherries; Others & Mixed; Strawberries; Raspberries; Blackcurrants; Other Soft Fruit	Fresh stone fruit, Other fresh soft fruit, Fresh melons, Other fresh fruit
Vegetables			
Brassica	0704 - Cabbages, cauliflowers, kohlrabi, kale and similar edible brassicas, fresh or chilled	Cauliflowers and Broccoli; Cabbages; Other Brassicas	Fresh cabbages, Fresh brussels sprouts, Fresh cauliflower
Legumes	0708 - Leguminous vegetables, shelled or unshelled, fresh or chilled	Beans; Peas; Dried Peas; Dried Chick Peas; Dried Beans	Fresh peas, Fresh beans
Lettuce	0705 - Lettuce "Lactuca sativa" and chicory "Cichorium spp.", fresh or chilled	Lettuce; Lettuce - protected veg	Leafy salads fresh (Lettuce and leafy salads, Prepared lettuce salads)
Onions	0703 - Onions, shallots, garlic, leeks and other alliaceous vegetables, fresh or chilled	Onions, Dry Bulb; Onions, Spring; Leeks; Garlic; Other Alliaceous; Onions	Fresh onions, leeks and shallots
Other vegetables	0707 - Cucumbers and gherkins, fresh or chilled; 0709 - Other vegetables, fresh or chilled	Asparagus; Baby leaf; Celery; Courgette; Cucumbers; Mushrooms; Others-protected veg; Others, field grown; Rhubarb; Self Blanching Celery; Sweet Peppers; Watercress; All Other Dried Vegetables; All Other Fresh Vegetables; Aubergines	Other fresh green vegetables, Fresh cucumbers, Fresh mushrooms, Miscellaneous fresh vegetables
Root vegetables	0706 - Carrots, turnips, salad beetroot, salsify, celeriac,	Beetroot; Carrots; Parsnips; Turnips and	Fresh carrots, Fresh turnips and swede, Other fresh root vegetables

For peer review only - http://bmjopen.bmj.com/site/about/guidelines.xhtml

	radishes and similar edible roots, fresh or chilled	Swedes; Carrots and Turnips	
Tomatoes	0702 - Tomatoes, fresh or chilled	Tomatoes	Fresh tomatoes
	Canned, dried, and frozen		
Fruits	0811 - Fruit and nuts, uncooked or cooked by steaming or boiling in water, frozen, whether or not containing added sugar or other sweetening matter; 0813 - Dried apricots, prunes, apples, peaches, pears, papaws "papayas", tamarinds and other edible fruits, and mixtures of edible and dried fruits or of edible nuts; 0814 - Peel of citrus fruit or melons, incl. watermelons, fresh, frozen, dried or provisionally preserved in brine, or in water with other additives	Approximated from "Other Fruits"	Tinned peaches, pears and pineapples; All other tinned or bottled fruit; Dried fruit; Frozen strawberries, apple slices, peach halves, oranges and other frozen fruits
Vegetables	0710 - Vegetables, uncooked or cooked by steaming or boiling in water, frozen; 0712 - Dried vegetables, whole, cut, sliced, broken or in powder, but not further prepared 0713 - Dried leguminous vegetables, shelled, whether or not skinned or split	Approximated from "Other Vegetables"	Tomatoes, canned or bottled; Peas, canned; Beans canned; Other canned vegetables; Dried pulses other than air-dried; Air-dried vegetables; Peas, frozen; Beans, frozen; Other frozen vegetables

For peer review only

Appendix B. Mortality projections.

We used a Bayesian Age-Period-Cohort (BAPC) model to estimate mortality projections between 2021 and 2030. The BAPC model attributes changes in mortality to (1) age effects, which capture physiological and social changes related to aging; (2) period effects, which are associated with environmental, scientific, and social changes related to calendar time of death; and (3) cohort effects, which describe generational differences due to year of birth. It then projects mortality probabilities by assuming that trends of these effects continue over time. The methodology has been previously described in detail.(5) Briefly, we used historic CHD and stroke mortality data stratified by sex and age (10-year groups from 25-34 until 85+) between 1981 and 2015 from the Office for National Statistics (ONS), defined using ICD-codes (Table B1). We also used historic population estimates (1985-2015) and population projections (2016-2030) by age and sex from the ONS. We applied the model using the BAMP statistical software which employs Markov Chain Monte Carlo simulations to estimate mortality projections. An age and sex specific ischemic to haemorrhagic stroke ratio (Table B2), estimated using 2016 mortality data by ICD-code from the ONS, was applied to overall stroke projections to estimate ischemic and haemorrhagic stroke mortality between 2021 and 2030, assuming no changes in clinical care over time. We combined ischemic and not specified stroke to better reflect evidence from the American Heart Association that suggests 87% of all stroke is ischemic.(6) Previous analyses in the UK have followed the same approach.(7)

Table B1. ICD codes for CVD outcomes

Type of CVD outcomes	ICD-9 codes (1981-2000)	ICD-10 codes (2011-2015 and 2016)
CHD	410-414	I20-I25
Overall stroke	430-438	I60-I69
Ischemic stroke		I63, I65-I67 (except I67.4)
Haemorrhagic stroke		I60-I62, I69.0-I69.2, I67.4
Other (not specified) stroke		I64, I69.4, I69.8

For peer review only - http://bmjopen.bmj.com/site/about/guidelines.xhtml

Table B2. Percentage of stroke deaths attributed to ischemic, haemorrhagic, and other (not specified) stroke and ischemic to haemorrhagic stroke ratio

Population group	Ischemic stroke	Haemorrhagic stroke	Other (not specified) stroke	Ischemic: Haemorrhagic*
Men 25-34	14%	82%	5%	0.22
Men 35-44	12%	79%	9%	0.26
Men 45-54	17%	67%	16%	0.50
Men 55-64	18%	53%	30%	0.90
Men 65-74	23%	37%	40%	1.71
Men 75-84	24%	28%	48%	2.57
Men 85+	30%	19%	51%	4.25
Women 25-34	23%	69%	9%	0.46
Women 35-44	14%	81%	5%	0.23
Women 45-54	14%	73%	13%	0.38
Women 55-64	15%	63%	23%	0.59
Women 65-74	18%	46%	36%	1.16
Women 75-84	23%	32%	46%	2.13
Women 85+	30%	17%	53%	4.98
Overall	*26%*	*28%*	*46%*	*2.58*

*Other (not specified) stroke was combined with ischemic stroke.

For peer review only

For peer review only - http://bmjopen.bmj.com/site/about/guidelines.xhtml

Appendix C. Effect of Brexit scenarios on price of F&V

Under each scenario, we assumed two main drivers of F&V price change. The first driver is the application of tariffs to imported commodities based on their country of origin. If there is a free trading agreement in place, these tariffs will be zero. However, if trade occurs under WTO regulations, the UK will have to apply import tariffs. These are required to be equal among all trading partners under the WTO's MFN clause that protects its members from discriminatory treatment. We assumed that the UK will adopt the EU MFN tariffs for all scenarios except scenario 4 (Liberalised regime) when the UK will reduce its MFN tariffs to zero.

The second driver of price change is attributed to an increase in transaction costs of EU trade due to post-Brexit border controls. We assumed that border controls will occur (1) to apply rules of origin and (2) to check technical requirements, such as Technical Barriers to Trade (TBT) and Sanitary and Phytosanitary (SPS) measures. Rules of origin determine commodities' country of origin and are necessary in order to apply appropriate trade restrictions such as tariffs. Within the Customs Union, such restrictions do not apply and goods move across borders freely. However, exiting the EU Customs Union will require checks for rules of origin for all commodities entering the UK from the EU. The cost of ensuring compliance with rules of origin regulations varies between different countries, depending on their trade arrangements, their infrastructure, the product traded, and other factors(8). A WTO Working Paper(8) suggests that administrative costs of compliance with rules of origin vary between 2% and 8%, recommending a 5% estimate as a commonly used benchmark.

In common with other free trading arrangements, the EU has integrated technical regulations that do not require border controls. However, when no free trading agreement is in place and trade occurs under WTO regulations, technical characteristics of commodities need to be checked at the border. Cadot et al. investigated the difference between the price-raising effect of SPS and TBT by commodity in the presence and absence of preferential arrangements that involve deep integration of technical requirements(9). They estimated a difference of 4.5% for vegetable products, which we used to approximate the increase in transaction costs of F&V under the Brexit scenarios that no preferential arrangement is assumed between the UK and the EU (Scenario 3 – No deal Brexit and Scenario 4 – Liberalised regime). However, this might overestimate the real effect as the analysis by Cadot et al. does not specify if the price-raising effects of SPS and TBT are due to transaction costs or demand increases reflecting the quality improvements that usually follow compliance with technical requirements.

For peer review only - http://bmjopen.bmj.com/site/about/guidelines.xhtml

Table C1 demonstrates the change in tariffs and transaction costs that will occur to F&V imports from the EU, non-EU countries under a preferential arrangement with the EU, and other non-EU countries, under each Brexit scenario.

Table C1. Added costs to F&V imports in each Brexit scenario

	**Imports from the EU**		**Duty-free imports from non-EU countries**	**Dutiable imports from non-EU countries**
	Tariffs	*Transaction costs*	*Tariffs*	*Tariffs*
No Brexit	*No tariffs*	*None*	*No tariffs*	*EU MFN tariffs*
**Scenario 1**	No tariffs	Rules of origin	EU MFN tariffs * 50%	EU MFN tariffs
**Scenario 2**	No tariffs	Rules of origin	EU MFN tariffs	EU MFN tariffs
**Scenario 3**	EU MFN tariffs	Rules of origin and technical requirements	EU MFN tariffs	EU MFN tariffs
**Scenario 4**	Zero MFN tariffs	Rules of origin and technical requirements	Zero MFN tariffs	Zero MFN tariffs

Scenario S1: Free trading agreement with the EU and with half of non-EU importers; Scenario S2: Free trading agreement with the EU and trading under WTO rules with non-EU importers; Scenario S3: No deal Brexit; Scenario S4: Liberalised trade regime

For peer review only

Appendix D. Estimating the effect of F&V intake change on mortality

The IMPACT Food Policy model estimates the proportional change in mortality due to change in the intake of each F&V subgroup by age and sex using the following formula:

$$MortalityChange = \left(1 - e^{beta \times IntakeChange}\right)$$

where *beta* is the natural logarithm of the relative risk between fruit or vegetable intake and CVD outcomes and *IntakeChange* is the change in intake of each F&V subgroup under the different scenarios.

The number of deaths attributable to each scenario for every F&V subgroup by age, sex, and year are then estimated as shown below:

$$Deaths = MortalityChange * Mortality$$

where *Mortality* is the counterfactual number of predicted deaths of CHD, ischemic stroke or haemorrhagic in each age-sex group in years 2021 to 2030.

In order to combine the effect of the different F&V subgroups into an overall F&V effect we used the cumulative risk-reduction approach as previously described by Bajekal et al.(10) Briefly, we estimated an adjustment factor as the ratio of an additive and a multiplicative effect as shown below:

$$AF = CR/AR$$

where

$$CR = 1 - \left(1 - abs(MortalityChange_1)\right) * \left(1 - abs(MortalityChange_2)\right) * \dots * \left(1 - abs(MortalityChange_i)\right)$$

and

$$AR = abs(MortalityChange_1) + abs(MortalityChange_2) + \cdots + abs(MortalityChange_i)$$

with *1, 2, …, i* being the different F&V subgroups.

We then estimated the overall attributable deaths as shown in the following equation

$$Deaths_{overall} = (Deaths_1 + Deaths_2 + \cdots + Deaths_i) * AF$$

where $Deaths_1$, $Deaths_2$, …, $Deaths_i$ are the number of deaths due to change in intake of each F&V subgroup.

For peer review only - http://bmjopen.bmj.com/site/about/guidelines.xhtml

Appendix E. Sensitivity analysis – substitution with canned, dried, and frozen F&V

We performed a sensitivity analysis in order to take into account substitution between fresh F&V and canned, dried, and frozen F&V. Trade data inputs for canned, dried, and frozen F&V and their classification using commodity codes are presented in Tables A1 and A2 respectively. Intake of canned, dried, and frozen F&V was estimated by applying a gradient of purchases from the Living Costs and Food Survey(11) to the overall F&V intake, similarly to F&V subgroups estimation (Table A2). We used own and cross-price elasticities for "Fresh fruits", "Fresh vegetables", "Tinned & dried fruit", and "Canned vegetables" by Tiffin et al. Frozen F&V were not included in the estimation of price elasticities.

For the model calculations, we first estimated the cross-price effect of the change in the price of fresh F&V on canned, dried, and frozen F&V using the appropriate cross-price elasticity. The effect of the different Brexit scenarios on price of overall fresh F&V was calculated as the ratio of the final changes in fresh F&V intake (after substitution between F&V subgroups was taken into account) to the own-price elasticities of fresh fruits and fresh vegetables.

We, then, estimated the effect of the different Brexit scenarios on prices of canned, dried, and frozen F&V, as described in Appendix B. Own and cross-price elasticities were employed to estimate the effect of the change in the price of canned, dried, and frozen F&V on the intake of canned, dried, and frozen F&V (own-price effect) and fresh F&V (cross-price effect), after the new equilibrium was reached. The overall intake change was the difference between baseline intake and intake when both prices of fresh F&V and canned, dried, and frozen F&V had changed. Effect of change in intake of fresh F&V on mortality was estimated as described in Appendix C.

For peer review only - http://bmjopen.bmj.com/site/about/guidelines.xhtml

Supplementary tables

Supplementary Table 1. Statistical distributions for model inputs and corresponding parameters used in the probabilistic sensitivity analysis.

Inputs	Distribution	Parameters	Source
Ad valorem EU MFN tariff (%)	Beta	Alpha=((1-mean)/variance - 1/mean)*mean^2; beta=alpha*(1/mean - 1) estimated from tariff averages and variance	Tariff Analysis Online, WTO(1)
Imports % supply	Beta	Alpha=((1-mean)/variance - 1/mean)*mean^2; beta=alpha*(1/mean - 1) estimated from the mean and variance of imports between 2006-2016	DEFRA(3)
Cost of rules of origin	Pert	mode: 5%; min: 2%; max: 8%	Abreu, 2013(8)
Cost of technical regulations	Pert	mode: 4.5%, max/min: +/-20%	Cadot, 2016(9)
Mortality	Pert	mode: death projections, best estimate; min: death projections, lower 95% credible limit; max: death projections, upper 95% credible limit	Own estimations using ONS data
Elasticities	Normal	Mean and SE using price elasticities between 2000 and 2009	Tiffin, 2011(12)
F&V intake	Gamma	alpha: mean^2/variance; beta: variance/mean	National Diet and Nutrition Survey Rolling Programme(13)
Relative Risks	Log normal	Parameters based on Barendregt, 2010(14)	Micha, 2017(15)

For peer review only

Supplementary Table 2. Estimated relative change in price of fruit and vegetable subgroups under each modelled Brexit scenario. Numbers in parentheses indicate 95% uncertainty intervals.

	Scenario 1	Scenario 2	Scenario 3	Scenario 4
Fruits				
Apples and Pears	2.9% (2.0%, 4.0%)	3.5% (2.5%, 4.6%)	9.1% (8.0%, 10.4%)	4.0% (3.0%, 5.1%)
Bananas	7.6% (7.4%, 7.9%)	14.8% (14.6%, 15.0%)	16.9% (16.6%, 17.1%)	0.9% (0.6%, 1.1%)
Citrus fruit	4.7% (2.7%, 7.8%)	6.8% (3.5%, 12.9%)	14.3% (7.6%, 27.2%)	4.2% (3.0%, 5.6%)
Grapes	3.5% (1.4%, 9.1%)	5.8% (1.7%, 16.9%)	8.7% (3.1%, 24.3%)	1.8% (0.1%, 2.7%)
Other fruits	3.2% (2.1%, 4.9%)	4.3% (2.5%, 7.3%)	9.0% (5.4%, 15.7%)	3.3% (2.1%, 4.4%)
Vegetables				
Brassica	1.5% (0.8%, 2.4%)	1.6% (0.8%, 2.5%)	6.5% (4.5%, 8.7%)	2.8% (1.8%, 4.1%)
Legumes	4.4% (3.0%, 5.9%)	7.8% (5.4%, 10.8%)	10.3% (7.1%, 13.9%)	1.4% (1.0%, 1.9%)
Lettuce	2.9% (1.7%, 4.3%)	2.9% (1.7%, 4.3%)	11.6% (9.6%, 13.9%)	5.5% (4.1%, 7.2%)
Onions	2.3% (1.4%, 3.3%)	2.5% (1.6%, 3.5%)	8.6% (7.4%, 9.9%)	3.5% (2.5%, 4.5%)
Other vegetables	3.4% (2.1%, 4.8%)	3.7% (2.3%, 5.1%)	12.3% (8.2%, 18.0%)	5.7% (4.3%, 7.2%)
Root vegetables	0.3% (0.1%, 0.5%)	0.3% (0.2%, 0.5%)	1.1% (0.6%, 1.7%)	0.4% (0.2%, 0.7%)
Tomatoes	3.8% (2.1%, 5.3%)	3.8% (2.1%, 5.3%)	14.9% (13.2%, 16.6%)	6.0% (4.4%, 7.7%)

Supplementary Table 3. Mean (standard deviation) intake of F&V (g/d) in England by age and sex, estimated using data from the National Diet and Nutrition Survey Rolling Programme Years 1-8.

Population group	Fruit intake	Vegetable intake
Men 25-44	86.5 (95.3)	187.9 (99.3)
Men 45-64	116.6 (114.0)	200.7 (109.0)
Men 65+	121.6 (111.8)	184.6 (101.8)
Women 25-44	93.3 (102.1)	184.8 (111.6)
Women 45-64	128.5 (118.1)	202.0 (115.0)
Women 65+	132.2 (117.1)	173.6 (86.3)
Overall	*111.1 (111.1)*	*190.0 (106.1)*

Means and standard deviation are weighted for non-response

For peer review only - http://bmjopen.bmj.com/site/about/guidelines.xhtml

For peer review only

Supplementary Table 4. Gradient of F&V subgroups intake, estimated using household purchase data (g/person/week) from the Family Food module of the Living Costs and Food Survey 2016/7.

F&V subgroup	Intake (%)
Apples & Pears	19.1
Bananas	25.5
Citrus fruit	16.1
Grapes	6.8
Other fruits	26.9
Canned, dried, and frozen fruits	*5.5*
Total fruits	***100.0***
Brassica	9.4
Legumes	2.0
Lettuce	4.5
Onions	10.4
Other vegetables	24.6
Root vegetables (excl. potatoes)	16.6
Tomatoes	7.7
Canned, dried, and frozen vegetables	*24.8*
Total vegetables	***100.0***

For peer review only

For peer review only - http://bmjopen.bmj.com/site/about/guidelines.xhtml

Supplementary Table 5. Estimated relative change in intake of F&V subgroups under each modelled Brexit scenario. Numbers in parentheses indicate 95% uncertainty intervals.

	Scenario 1	Scenario 2	Scenario 3	Scenario 4
Fruits	***-4.4% (-5.2%, -3.8%)***	***-7.0% (-8.4%, -5.9%)***	***-11.4% (-14.2%, -9.5%)***	***-2.8% (-3.3%, -2.3%)***
Apples and Pears	-3.3% (-4.1%, -2.5%)	-4.4% (-5.4%, -3.5%)	-9.4% (-10.7%, -8.2%)	-3.5% (-4.3%, -2.7%)
Bananas	-5.4% (-5.8%, -5.0%)	-10% (-10.8%, -9.3%)	-12.1% (-13.2%, -11.2%)	-1.1% (-1.4%, -0.9%)
Citrus fruit	-4.4% (-7.1%, -2.7%)	-6.4% (-11.6%, -3.6%)	-13.0% (-24%, -7.3%)	-3.8% (-5%, -2.7%)
Grapes	-3.3% (-6.7%, -2.0%)	-5.4% (-11.9%, -3.0%)	-8.2% (-17.3%, -4.9%)	-1.8% (-2.4%, -0.7%)
Other fruits	-4.3% (-5.7%, -3.3%)	-6.2% (-8.7%, -4.7%)	-11.4% (-16.8%, -8.3%)	-3.6% (-4.6%, -2.5%)
Vegetables	***-2.5% (-3.1%, -1.9%)***	***-2.7% (-3.3%, -2.2%)***	***-9.1% (-11.0%, -7.8%)***	***-4.0% (-4.6%, -3.4%)***
Brassica	-1.5% (-2.2%, -1.0%)	-1.5% (-2.2%, -0.9%)	-6.4% (-8.1%, -4.9%)	-2.8% (-3.7%, -2.0%)
Legumes	-3.3% (-4.5%, -2.3%)	-5.7% (-7.8%, -4.0%)	-8.3% (-11%, -6.1%)	-1.6% (-2.1%, -1.1%)
Lettuce	-2.7% (-3.7%, -1.8%)	-2.9% (-3.9%, -2.0%)	-10.2% (-11.8%, -8.7%)	-4.6% (-5.9%, -3.6%)
Onions	-2.2% (-3%, -1.5%)	-2.4% (-3.2%, -1.7%)	-8.3% (-9.5%, -7.3%)	-3.5% (-4.3%, -2.7%)
Other vegetables	-3.7% (-5.0%, -2.6%)	-4.1% (-5.5%, -2.9%)	-13.4% (-18.6%, -9.7%)	-6.1% (-7.5%, -4.9%)
Root vegetables	-0.9% (-1.1%, -0.6%)	-0.9% (-1.2%, -0.6%)	-3.5% (-4.2%, -2.8%)	-1.5% (-1.8%, -1.2%)
Tomatoes	-3% (-4.1%, -1.9%)	-3.1% (-4.2%, -2%)	-11.8% (-13.1%, -10.6%)	-4.8% (-5.9%, -3.6%)

For peer review only

For peer review only - http://bmjopen.bmj.com/site/about/guidelines.xhtml

Supplementary Table 6. Estimated number of counterfactual (95% credible interval) and attributable (95% uncertainty interval) CHD and stroke deaths and mortality increase (%) in 2030, by age and sex, under each modelled Brexit scenario.

		Scenario 1	Scenario 2	Scenario 3	Scenario 4
CVD	Counterfactual deaths (2030)	71120 (38140, 164210)	71120 (38140, 164210)	71120 (38140, 164210)	71120 (38140, 164210)
	Attributable deaths (2030)	450 (250, 810)	630 (340, 1,180)	1,340 (780, 2,350)	450 (270, 750)
	Mortality reduction (%)	0.6%	0.9%	1.9%	0.6%
CHD	Counterfactual deaths (2030)	43110 (27160, 73740)	43110 (27160, 73740)	43110 (27160, 73740)	43110 (27160, 73740)
	Attributable deaths (2030)	140 (80, 230)	200 (110, 340)	420 (260, 690)	140 (90, 210)
	Mortality reduction (%)	0.3%	0.5%	1.0%	0.3%
Stroke	Counterfactual deaths (2030)	28010 (10970, 90470)	28010 (10970, 90470)	28010 (10970, 90470)	28010 (10970, 90470)
	Attributable deaths (2030)	320 (180, 590)	440 (240, 860)	960 (550, 1720)	330 (190, 570)
	Mortality reduction (%)	1.1%	1.5%	3.3%	1.1%

For peer review only

Supplementary Table 7. Estimated relative change in price of fresh and canned, dried, and frozen F&V under each modelled Brexit scenario. Numbers in parentheses indicate 95% uncertainty intervals.

	Scenario 1	Scenario 2	Scenario 3	Scenario 4
Fresh fruits	4.4%<br>(3.8%, 5.2%)	7.1%<br>(6%, 8.5%)	11.6%<br>(9.6%, 14.4%)	2.9%<br>(2.4%, 3.3%)
Canned, dried, and frozen fruits	3.4%<br>(2.1%, 5%)	4.1%<br>(2.4%, 6.9%)	10.3%<br>(5.7%, 20.0%)	4%<br>(2%, 5.6%)
Fresh vegetables	2.5%<br>(2%, 3%)	2.7%<br>(2.2%, 3.3%)	9.2%<br>(7.8%, 11%)	4%<br>(3.4%, 4.6%)
Canned, dried, and frozen vegetables	2.4%<br>(1.4%, 3.3%)	2.6%<br>(1.5%, 3.9%)	6.8%<br>(3.9%, 16.7%)	3.1%<br>(-1.2%, 4.8%)

Supplementary Table 8. Estimated relative change in intake of fresh and canned, dried, and frozen F&V under each modelled Brexit scenario. Numbers in parentheses indicate 95% uncertainty intervals.

	Scenario 1	Scenario 2	Scenario 3	Scenario 4
Fresh fruits	-4.5%<br>(-5.3%, -3.8%)	-7.1%<br>(-8.5%, -6.0%)	-11.7%<br>(-14.4%, -9.7%)	-2.9%<br>(-3.4%, -2.4%)
Canned, dried, and frozen fruits	-2.4%<br>(-3.7%, -1.3%)	-2.8%<br>(-5.0%, -1.3%)	-7.3%<br>(-15.3%, -3.8%)	-3.0%<br>(-4.2%, -1.4%)
Fresh vegetables	-2.6%<br>(-3.2%, -2.1%)	-2.9%<br>(-3.5%, -2.3%)	-9.6%<br>(-11.4%, -8.2%)	-4.1%<br>(-4.8%, -3.6%)
Canned, dried, and frozen vegetables	-1.0%<br>(-1.6%, -0.4%)	-1.1%<br>(-1.9%, -0.4%)	-2.6%<br>(-8.5%, -0.7%)	-1.2%<br>(-2.3%, 1.4%)

For peer review only

For peer review only - http://bmjopen.bmj.com/site/about/guidelines.xhtml

Supplementary Table 9. Estimated number of cumulative CVD deaths and 95% uncertainty intervals for 2021-2030 associated with each modelled Brexit scenario – Sensitivity analyses

Scenario	Coronary heart disease	Stroke	Cardiovascular disease
Sensitivity Analysis A: Taking into account effect of canned, dried, and frozen F&V			
Scenario 1	1,400 (760, 2,830)	2,860 (1,440, 5,590)	4,260 (2,200, 8,420)
Scenario 2	1,970 (1,010, 4,190)	3,980 (1,940, 8,170)	5,950 (2,940, 12,360)
Scenario 3	4,230 (2,370, 8,070)	8,760 (4,630, 16,720)	12,990 (7,000, 24,790)
Scenario 4	1,390 (830, 2,420)	2,920 (1,610, 5,200)	4,310 (2,430, 7,620)
Sensitivity Analysis B: Increasing domestic production by 2% a year*			
Scenario 1	1,300 (690, 2,550)	2,590 (1,310, 5,190)	3,890 (2,000, 7,730)
Scenario 2	1,850 (930, 3,860)	3,650 (1,790, 7,620)	5,500 (2,720, 11,470)
Scenario 3	3,890 (2,190, 7,300)	7,800 (4,090, 14,880)	11,680 (6,280, 22,180)
Scenario 4	1,260 (740, 2,150)	2,590 (1,420, 4,700)	3,860 (2,150, 6,850)

**Increase in domestic supply was assumed for all F&V excluding bananas, citrus fruits, and grapes.*

Supplementary Table 10. Estimated relative change in intake of F&V in the first year (2021) and last year (2030) of the modelling period under each modelled Brexit scenario, assuming that domestic supply of F&V will increase by approximately 2% a year*. Numbers in parentheses indicate 95% uncertainty intervals.

Scenario	Change in intake	
	Fruits	Vegetables
2021		
Scenario 1	-4.4% (-5.2%, -3.8%)	-2.5% (-3.0%, -1.9%)
Scenario 2	-7.0% (-8.4%, -5.9%)	-2.7% (-3.3%, -2.2%)
Scenario 3	-11.4% (-14.2%, -9.5%)	-9.1% (-11.0%, -7.8%)
Scenario 4	-2.8% (-3.3%, -2.3%)	-4.0% (-4.6%, -3.4%)
2030		
Scenario 1	-4.1% (-4.9%, -3.5%)	-2.1% (-2.5%, -1.6%)
Scenario 2	-6.6% (-8.0%, -5.7%)	-2.3% (-2.7%, -1.8%)
Scenario 3	-10.7% (-13.3%, -8.9%)	-7.6% (-9.2%, -6.5%)
Scenario 4	-2.5% (-2.9%, -2.1%)	-3.3% (-3.8%, -2.8%)

**Increase in domestic supply was assumed for all F&V excluding bananas, citrus fruits, and grapes.*

For peer review only

For peer review only - http://bmjopen.bmj.com/site/about/guidelines.xhtml

References

1. WTO. Tariff Analysis Online [Available from: http://tao.wto.org/default.aspx.

2. HM Revenue & Customs. UK Trade Statistics [Available from: https://www.uktradeinfo.com/.

3. Department for Environment Food & Rural Affairs (DEFRA). Horticulture Statistics 2016. 2017 [Available from: https://www.gov.uk/government/collections/horticultural-statistics.

4. World Bank World Integrated Trade Solution (WITS) [Available from: https://wits.worldbank.org/default.aspx.

5. Guzman Castillo M, Gillespie DOS, Allen K, Bandosz P, Schmid V, Capewell S, et al. Future Declines of Coronary Heart Disease Mortality in England and Wales Could Counter the Burden of Population Ageing. PLoS One. 2014;9(6):e99482.

6. Benjamin EJ, Virani SS, Callaway CW, Chang AR, Cheng S, Chiuve SE, et al. Heart Disease and Stroke Statistics—2018 Update: A Report From the American Heart Association. Circulation. 2018.

7. Brownrigg JRW, Hughes CO, Burleigh D, Karthikesalingam A, Patterson BO, Holt PJ, et al. Microvascular disease and risk of cardiovascular events among individuals with type 2 diabetes: a population-level cohort study. The Lancet Diabetes & Endocrinology. 2016;4(7):588-97.

8. Abreu MD. Preferential rules of origin in regional trade agreements. World Trade Organization, Economic Research and Statistics Division; 2013.

9. Cadot O, Gourdon J. Non-tariff measures, preferential trade agreements, and prices: new evidence. Rev World Econ. 2016;152(2):227-49.

10. Bajekal M, Scholes S, Love H, Hawkins N, O'Flaherty M, Raine R, et al. Analysing Recent Socioeconomic Trends in Coronary Heart Disease Mortality in England, 2000-2007: A Population Modelling Study. PLoS Med. 2012;9(6):e1001237.

11. Office for National Statistics, Department for Environment Food and Rural Affairs. Living Costs and Food Survey, 2016-2017. UK Data Service; 2018

12. Tiffin R, Balcombe K, Salois M, Kehlbacher A. Estimating Food and Drink Elasticities 2011 [Available from: https://www.gov.uk/government/uploads/system/uploads/attachment_data/file/137726/defra-stats-foodfarm-food-price-elasticities-120208.pdf.

13. NatCen Social Research, MRC Elsie Widdowson Laboratory, University College London. Medical School. National Diet and Nutrition Survey Years 1-8, 2008/09-2015/16. 9th Edition ed: UK Data Service; 2018

14. Barendregt JJ. The effect size in uncertainty analysis. Value Health. 2010;13(4):388-91.

15. Micha R, Peñalvo JL, Cudhea F, Imamura F, Rehm CD, Mozaffarian D. Association between dietary factors and mortality from heart disease, stroke, and type 2 diabetes in the united states. JAMA. 2017;317(9):912-24.

For peer review only - http://bmjopen.bmj.com/site/about/guidelines.xhtml

BMJ Open

Impacts of Brexit on fruit and vegetable intake and cardiovascular disease in England: a modelling study

Journal:	*BMJ Open*
Manuscript ID	bmjopen-2018-026966.R1
Article Type:	Research
Date Submitted by the Author:	12-Dec-2018

Complete List of Authors:	Seferidi, Paraskevi; Imperial College London School of Public Health, Public Health Policy Evaluation Unit<br>Laverty, Anthony; Imperial College London School of Public Health, Public Health Policy Evaluation Unit<br>Pearson-Stuttard, Jonny; Imperial College London School of Public Health<br>Bandosz, Piotr; University of Liverpool, Department of Public Health & Policy<br>Collins, Brendan; University of Liverpool, Department of Public Health and Policy<br>Castillo, Maria; University of Liverpool, Public Health and Policy<br>Capewell, Simon; University of Liverpool, Department of Public Health<br>O'Flaherty, M; University of Liverpool, Department of Public Health<br>Millett, Christopher; Imperial College London School of Public Health, Public Health Policy Evaluation Unit

**Primary Subject Heading**:	Public health
Secondary Subject Heading:	Nutrition and metabolism
Keywords:	Brexit, fruits and vegetables, cardiovascular disease, trade policy, diet

SCHOLARONE™
Manuscripts

IMPACTS OF BREXIT ON FRUIT AND VEGETABLE INTAKE AND CARDIOVASCULAR DISEASE IN ENGLAND: A MODELLING STUDY

Paraskevi Seferidi[1], Anthony A Laverty[1], Jonathan Pearson-Stuttard[1,2], Piotr Bandosz[2,3], Brendan Collins[2], Maria Guzman-Castillo[2], Simon Capewell[2], Martin O'Flaherty[2], Christopher Millett[1]

[1]Public Health Policy Evaluation Unit, School of Public Health, Imperial College London, London, UK [2]Department of Public Health and Policy, University of Liverpool, Liverpool, UK; [3]Department of Preventive Medicine and Education, Medical University of Gdańsk, Gdańsk, Poland

Correspondence to: P Seferidi, paraskevi.seferidi14@imperial.ac.uk, Public Health Policy Evaluation Unit, School of Public Health, Imperial College London, Reynolds Building, St Dunstan's Road, W6 8RP, London, UK

Word count: 3,769

For peer review only

For peer review only - http://bmjopen.bmj.com/site/about/guidelines.xhtml

ABSTRACT

Objectives

To estimate the potential impacts of different Brexit trade policy scenarios on the price and intake of fruits and vegetables (F&V) and consequent cardiovascular disease (CVD) deaths in England between 2021 and 2030.

Design

Economic and epidemiological modelling study with probabilistic sensitivity analysis.

Setting

The model combined publicly available data on F&V trade, published estimates of UK-specific price elasticities, national survey data on F&V intake, estimates on the relationship between F&V intake and CVD from published meta-analyses, and CVD mortality projections for 2021-2030.

Participants

English adults aged 25 years and older.

Interventions

We modelled four potential post-Brexit trade scenarios: (1) Free trading agreement with the EU and maintaining half of non-EU free trade partners; (2) Free trading agreement with the EU but no trade deal with any non-EU countries; (3) No deal Brexit; (4) Liberalised trade regime which eliminates all import tariffs.

Outcome measures

Cumulative coronary heart disease and stroke deaths attributed to the different Brexit scenarios modelled between 2021 and 2030.

Results

Under all Brexit scenarios modelled, prices of F&V would increase, especially for those highly dependent on imports. This would decrease intake of F&V between 2.5% (95% Uncertainty Interval: 1.9% to 3.1%) and 11.4% (9.5% to 14.2%) under the different scenarios. Our model suggests that a no deal Brexit scenario would be the most harmful, generating approximately 12,400 (6,690 to 23,390) extra CVD deaths between 2021 and 2030, whereas establishing a free trading agreement with the EU would have a lower impact on mortality, contributing approximately 5,740 (2,860 to 11,910) extra CVD deaths.

For peer review only - http://bmjopen.bmj.com/site/about/guidelines.xhtml

Conclusions

Trade policy under all modelled Brexit scenarios could increase price and decrease intake of F&V, generating substantial additional CVD mortality in England. The UK government should consider the population health implications of Brexit trade policy options, including changes to food systems.

ARTICLE SUMMARY

Strengths and limitations of this study

- This study uses nationally representative dietary intake data, robust mortality projections and both own- and cross-price effect estimates to evaluate the impact of different Brexit scenarios on the price and intake of disaggregated fruit and vegetable subgroups and associated CVD outcomes.
- Fruit and vegetable imports were approximated using the latest available data, assuming import shares will not change after Brexit.
- Trade transaction costs due to technical regulations are based on published estimates that might also reflect costs attributed to demand changes thus overestimating real transaction costs.
- Response to price increases of fruits and vegetables were estimated using historical purchase data and might not reflect consumer response to price change post-Brexit.
- The scenarios modelled are not exhaustive and more trade policy options might arise as Brexit negotiations continue.

For peer review only

INTRODUCTION

Trade policy exerts a powerful influence on risk factors for non-communicable disease and thus for population health.[1] One prominent mechanism is the effect of trade on food environments, through changes in the availability and affordability of food commodities.[2] Evidence suggests that entering new trading blocs and opening domestic markets to international trade has been associated with increased imports, sales, and intake of highly processed foods.[3-6] However, there is a lack of evidence on the effect of trade on healthy food commodities, while no previous studies have investigated the potential impacts of exiting a large trading bloc.

Brexit – the impending exit of the United Kingdom (UK) from the European Union (EU) – will have profound implications across a range of sectors. One of the main changes will involve the introduction of a new trade regime that will redefine the trade relationships between the UK and the EU and non-EU countries. This is likely to affect food environments in the UK, given its high dependency on imports to meet its dietary needs.[7] This is particularly the case for fruits and vegetables (F&V). For example, in 2017, 84% of fruits and 43% of vegetables in the UK were imported.[8] Thus, changes to the UK trade regime are likely to affect prices of F&V by increasing costs of trade. With price being one of the main determinants of consumer behaviour in the UK,[9] Brexit could affect F&V intake with significant implications for UK diets.

Low F&V intake is a major risk factor of many non-communicable diseases, including cardiovascular disease (CVD). Approximately 1.3 million CVD deaths could have been prevented globally in 2013 if intake of F&V was higher than 500 grams/day (g/d).[10] The UK performs poorly in terms of F&V intake; only 27% of adults aged 19-64 years and 35% of those above 65 years achieve daily recommended intakes.[11] Thus, Brexit trade scenarios that change the price and availability of F&V are likely to have substantial impacts on CVD outcomes in the UK. This analysis aimed to quantify the potential impacts of F&V price changes due to Brexit on CVD deaths in England between 2021 and 2030.

METHODS

We extended the previously validated IMPACT Food Policy model[12-14] to estimate the effect of different Brexit scenarios on the price and intake of F&V in England. We then modelled consequent impacts on cumulative coronary heart disease (CHD) and stroke mortality in English adults aged 25 and above, stratified by age and sex, over a ten year period in order to capture medium-term effects.

For peer review only - http://bmjopen.bmj.com/site/about/guidelines.xhtml

Data sources

We used import and tariff data from the World Trade Organization (WTO)[15] and the HM Revenues and Customs (HMRC)[16] to estimate the effect of different Brexit scenarios on price of F&V. We describe these data in more detail in Appendix A.

The effect of price changes on intake of F&V products was estimated using published UK-specific price elasticities.[17] Price elasticities measure the change in demand of a good as a response to a change in its own price (own-price elasticity) or the price of another good (cross-price elasticity). Price elasticities for five fruit (Apples and Pears; Bananas; Citrus fruit; Grapes; Other fruits) and seven vegetable (Brassica, such as cabbages, cauliflower, and broccoli; Legumes, such as beans and peas; Lettuce; Onions; Other vegetables; Root vegetables; Tomatoes) subgroups were available. We used conditional uncompensated own and cross-price elasticities to take into account the differential effect of Brexit on the prices of different F&V. Conditional price elasticities, usually recommended at this level of product disaggregation, assume that food expenditure available to all other products remains constant, and uncompensated price elasticities assume that consumer income is held constant.[17]

We estimated total F&V intake in English adults by sex and age (25-44, 45-74, 75+) using data from the National Diet and Nutrition Survey Rolling Programme (2008/09-2015/16).[18] This is a nationally representative survey of UK adults and children that uses a four-day food diary to evaluate dietary intake. As the effect of the different Brexit scenarios varies across different F&V products, we used household purchase data (in grams/person/week) for England from the Family Food Module of the Living Costs and Food Survey 2016/17[19] to approximate intake of F&V subgroups. The survey uses a two-week diary to identify purchases and expenditures of foods in UK households. We adjusted the overall F&V intake using the ratio of purchases of F&V subgroups with overall F&V purchases. We assumed a constant ratio across all sex and age groups.

Changes in F&V intake were translated into mortality changes using relative risk estimates of CHD, ischemic stroke, and haemorrhagic stroke with fruit intake and vegetable intake, derived from meta-analyses of longitudinal studies and further adjusted for an effect modification by age.[20] Employing age-specific relative risks allowed us to incorporate the declining effect of age on the relationships between risk factors and CVD, which has been previously described.[21] The authors assumed a linear relationship between risk and mortality of the outcomes, i.e. associations with incident CVD and CVD mortality were used interchangeably. The relative risk estimates are presented in Supplementary Table 1.

For peer review only

For peer review only - http://bmjopen.bmj.com/site/about/guidelines.xhtml

Finally, this model employed sex and age (10-year groups from 25-34 until 85+) specific CVD mortality projections between 2021 and 2030 which were estimated using a Bayesian Age-Period-Cohort model and historic CVD mortality and population data from the Office for National Statistics. The methodology is described in more detail in Appendix B.

The data and data sources used in this model are summarised in Table 1.

Table 1. Model inputs and data sources

Data	Data source
Import and tariff data	World Trade Organisation[15] and HM Revenues and Customs[16]
Supply data (domestic production, imports, and exports)	Department for Environment, Food and Rural Affairs[22]
Price elasticities (mean of annual elasticity estimates between 2000-2009)	Tiffin, 2011[17]
Means of F&V intake by age and sex	National Diet and Nutrition Survey Rolling Programme Years 1-8 (2008/09-2015/16)[18]
F&V subgroups gradient (ratio to overall F&V intake)	Purchase data from the Living Costs and Food Survey 2016/17[19]
Relative Risk for CHD/ ischemic stroke/ haemorrhagic stroke by serving of fruit/vegetable intake by age	Micha, 2017[20]
CHD and stroke mortality projections for England by age and sex (2021-2030)	Own estimations using historic mortality data and population projections from the Office for National Statistics
Ischemic to Haemorrhagic stroke ratio, estimated using number of deaths by ICD-code, age, and sex in 2016	Office for National Statistics

Modelled scenarios

The UK, as an EU Member State, is part of the EU Single Market and Customs Union. As a result, trade between the UK and the EU is tariff-free and frictionless with no tariff or non-tariff trade barriers in place.[23] Trade between the UK and non-EU countries is also regulated by the EU, with common preferential trade agreements and external tariffs across all EU Member States. At an international level, trade is regulated by the WTO which is used as a platform for its members to negotiate and ratify trade agreements.

Following the referendum in June 2016, the UK government has announced its intentions to leave the EU Single Market and Customs Union and negotiate a new trading relationship with the EU and third countries.[24] The current intention from both the UK and the EU is that this will happen two

For peer review only - http://bmjopen.bmj.com/site/about/guidelines.xhtml

years after Brexit, following a transition period that will maintain the trade status quo while allowing the UK to negotiate a future permanent trading position.

For this analysis, we modelled four post-Brexit trade scenarios commencing in 2021 when the transition period will end:

1. Free trading agreement with the EU and third countries (S1).
   In this scenario, the UK establishes a zero-tariff free trading agreement with the EU while it also maintains half of its non-EU tariff-free F&V importers.
2. Free trading agreement with the EU (S2).
   Under this scenario, the UK establishes a zero-tariff free trading agreement with the EU but loses all preferential access to markets of non-EU F&V importers.
3. No deal Brexit (S3).
   Under a no deal scenario the UK falls back into a WTO default position, having no preferential arrangements with the EU or non-EU importers.
4. Liberalised trade regime (S4).
   Under a liberalised trade regime, the UK trades under WTO regulations with no specific trade arrangements in place, but eliminates all its F&V import tariffs. This is an extreme case scenario that shows the impact of non-tariff trade barriers on F&V trade.

Under each scenario, we assumed two main drivers of F&V import price change. First, we expect an increase in transaction costs when trading with the EU due to a rise in post-Brexit border controls. We assumed a 5% increase in transaction costs due to rules of origin checks[25] in the scenarios that a free trading agreement is applied and an additional 4.5% increase due to checks for technical regulations in the scenarios that no preferential trade arrangements are in place.[26] We describe these estimates and its sources in detail in Appendix C. Second, we assumed changes in F&V import tariffs[15] that vary under the different Brexit scenarios. Specifically, import tariffs were assumed to be zero under a free trading agreement or a liberalised regime and equal to the EU tariffs otherwise. Table 2 demonstrates the change in tariffs and transaction costs that would occur to F&V imports from the EU, non-EU countries under a preferential arrangement with the EU, and other non-EU countries, under each Brexit scenario. Calculations for each scenario are described in more detail in Appendix C. The estimated change in price of F&V imports was translated into change in wholesale F&V price using an estimate of imports as a percentage of overall F&V supply in the UK, obtained from the Department for Environment, Food & Rural Affairs (DEFRA)[22] (see Appendix A for more details).

For peer review only - http://bmjopen.bmj.com/site/about/guidelines.xhtml

Table 2. Added costs to F&V imports in each Brexit scenario

	Imports from the EU		Duty-free imports from non-EU countries	Dutiable imports from non-EU countries
	Tariffs	*Transaction costs*	*Tariffs*	*Tariffs*
No Brexit	*No tariffs*	*None*	*No tariffs*	*EU MFN tariffs*
**Scenario 1**	No tariffs	Rules of origin	EU MFN tariffs*50%	EU MFN tariffs
**Scenario 2**	No tariffs	Rules of origin	EU MFN tariffs	EU MFN tariffs
**Scenario 3**	EU MFN tariffs	Rules of origin and technical regulations	EU MFN tariffs	EU MFN tariffs
**Scenario 4**	Zero MFN tariffs	Rules of origin and technical regulations	Zero MFN tariffs	Zero MFN tariffs

Scenario 1: Free trading agreement with the EU and third countries; Scenario 2: Free trading agreement with the EU; Scenario 3: No deal Brexit; Scenario 4: Liberalised trade regime; MFN: Most-Favoured-Nation (see Appendix C for more details)

The IMPACT Food Policy model

The IMPACT Food Policy model has been previously used to estimate the effect of different policies on dietary intake and CVD in the UK[12] and elsewhere.[27] We extended the model to estimate the effect of different Brexit scenarios on prices of F&V subgroups as described above. We then translated changes in prices into changes in intake taking into account potential substitution between subgroups. We allowed the price of each F&V subgroup to change one by one until final equilibrium was reached, i.e. when prices of all F&V subgroups had changed. The intake change was the difference between intake at final equilibrium and baseline. Finally, the model translated changes in intake into changes in mortality using appropriate relative risks. We assumed common relative risks for all fruit and all vegetable subgroups. The risk reduction effect of overall F&V was estimated using a cumulative risk-reduction approach.[28] Changes in mortality were expressed as number of deaths attributed to each Brexit scenario. CVD mortality was estimated as the sum of CHD, ischemic stroke, and haemorrhagic stroke. Based on recent evidence,[29] we assumed a lag time of less than a year for changes in F&V intake to impact CVD outcomes, thus lag time was not explicitly incorporated in the model. Calculations are described in more detail in Appendix D and a schematic representation of the model is shown in Supplementary Figure 1.

Uncertainty and sensitivity analyses

We performed a probabilistic sensitivity analysis to take into account uncertainty of model inputs. We employed Monte Carlo simulations to repeatedly draw random values of the inputs from their statistical distributions and run the model across multiple iterations. The estimates and their respective statistical distributions are presented in Supplementary Table 2. The median and 2.5 and

For peer review only - http://bmjopen.bmj.com/site/about/guidelines.xhtml

97.5 percentiles of 1,000 iterations were used to produce estimates and their 95% uncertainty intervals (95% UI).

As a response to an increase in the price of F&V, consumers may shift to other F&V alternatives. We performed an additional sensitivity analysis to take into account possible substitution between fresh F&V and canned, dried, and frozen F&V. The effect of the different Brexit scenarios on price of canned, dried, and frozen F&V was estimated following the same methodology as the main analysis. Then, cross-price elasticities were used to identify the effect of change in the price of canned, dried, and frozen F&V on fresh F&V. Finally, the effect of change in intake of fresh F&V on CVD mortality was calculated. As the import and purchase data used in this analysis did not allow for disaggregation of canned, dried, and frozen F&V based on their content in additives, such as salt or sugar, we conservatively assumed that canned, dried, and frozen F&V were not associated with CVD outcomes. Details about data sources and calculations are presented in Appendix E.

Finally, we performed a second additional sensitivity analysis that allows domestic production of F&V to increase as a response to the increase in price of imported F&V. We assumed a 2% increase per year for all F&V subgroups except bananas, citrus fruits, and grapes which have currently no domestic production in the UK.

Patient and public involvement

There was no patient and public involvement in this study.

RESULTS

F&V prices

The estimated effect of Brexit scenarios on price of F&V subgroups are presented in Figure 1. Price effects varied among different F&V subgroups, depending on the origin of imports, the applied tariffs, and the percentage of supply that is imported.

The no deal Brexit scenario (S3) would affect prices of F&V the most, especially for F&V that are highly dependent on imports. For example, the model suggested that under a no deal Brexit scenario, prices would increase by 16.9% (95% UI: 16.6% to 17.1%) for bananas, 14.3% (7.6% to 27.2%) for citrus fruit, and 14.9% (13.2% to 16.6%) for tomatoes. In contrast, prices of root vegetables, whose imports contribute only 5% to their total supply, would increase between 0.3% (0.1% to 0.5%) and 1.1% (0.6% to 1.7%) under different Brexit scenarios. While a liberalised trade regime (S4) will eliminate all import tariffs, it would still increase prices of F&V due to the effect of non-tariff trade barriers on EU trade. Thus, it would mostly affect prices of F&V that are primarily imported from the EU, like citrus fruits and tomatoes with an estimated 4.2% (3.0% to 5.6%) and

For peer review only - http://bmjopen.bmj.com/site/about/guidelines.xhtml

6.0% (4.4% to 7.7%) increase in price respectively. More detailed price effects are presented in Supplementary Table 3.

F&V intake

We estimated that mean intake of F&V at baseline was 111 g/d and 190 g/d respectively (Supplementary Table 4). The most consumed fruits were bananas (26% of fruit intake) and apples and pears (19% of fruit intake), while root vegetables were the most consumed vegetables (17% of vegetable intake) (Supplementary table 5).

We estimated that intake of F&V would reduce under all Brexit scenarios (Table 3). The no deal Brexit scenario (S3) would have the largest effect, reducing intake of fruits by approximately 11.4% (9.5% to 14.2%) and vegetables by approximately 9.1% (7.8% to 11.0%). If a free trading agreement with the EU is established (S2), vegetable intake would reduce by 2.7% (2.2% to 3.3%), whereas intake of fruits would reduce by 7.0% (5.9% to 8.4%). Intake effects on F&V subgroups are presented in Supplementary Table 6.

Table 3. Estimated relative change in intake of F&V under each modelled Brexit scenario.

Scenario	Change in intake (95% UI)	
	Fruits	Vegetables
Scenario 1	-4.4% (-5.2% to -3.8%)	-2.5% (-3.1% to -1.9%)
Scenario 2	-7.0% (-8.4% to -5.9%)	-2.7% (-3.3% to -2.2%)
Scenario 3	-11.4% (-14.2% to -9.5%)	-9.1% (-11.0% to -7.8%)
Scenario 4	-2.8% (-3.3% to -2.3%)	-4.0% (-4.6% to -3.4%)

Scenario 1: Free trading agreement with the EU and third countries; Scenario 2: Free trading agreement with the EU; Scenario 3: No deal Brexit; Scenario 4: Liberalised trade regime

CVD mortality

The number of deaths attributable to each Brexit scenario are presented in Table 4 and Figure 2. The no deal Brexit scenario (S3) would be the most impactful, contributing approximately 12,400 (6,690 to 23,390) extra CVD deaths or a 1.7% increase in CVD mortality over the modelling period of 2021-2030. Establishing a free trading agreement with the EU (S2) would have lower impacts on mortality contributing approximately 5,740 (2,860 to 11,910) extra CVD deaths or a 0.8% increase in CVD mortality over the ten-year period. Free trading agreements with both the EU and half of the UK's F&V importers (S1) would have more attenuated impacts, contributing approximately 4,110 (2,130 to 8,100) extra CVD deaths or a 0.6% increase in CVD mortality. Even under a liberalised regime that eliminates all import tariffs (S4), Brexit would contribute approximately 4,160 (2,330 to 7,380) extra CVD deaths or a 0.6% increase in CVD mortality between 2021 and 2030 due to an increase in

For peer review only - http://bmjopen.bmj.com/site/about/guidelines.xhtml

transaction costs of EU imports. Supplementary Table 7 provides the estimated effects on CVD mortality for a single year of the modelling analysis.

Table 4. Estimated number of cumulative CVD deaths and 95% UI for 2021-2030 associated with each modelled Brexit scenario

Scenario	Coronary heart disease	Stroke	Cardiovascular disease
Scenario 1	1,360 (730 to 2,670)	2,740 (1,400 to 5,430)	4,110 (2,130 to 8,100)
Scenario 2	1,930 (980 to 3,990)	3,810 (1,880 to 7,910)	5,740 (2,860 to 11,910)
Scenario 3	4,110 (2,330 to 7,660)	8,290 (4,360 to 15,730)	12,400 (6,690 to 23,390)
Scenario 4	1,360 (790 to 2,300)	2,810 (1,540 to 5,080)	4,160 (2,330 to 7,380)

Scenario 1: Free trading agreement with the EU and third countries; Scenario 2: Free trading agreement with the EU; Scenario 3: No deal Brexit; Scenario 4: Liberalised trade regime

Sensitivity Analyses

Our model suggested that prices of canned, dried, and frozen F&V would increase by approximately 10.3% (5.7% to 20.0%) for fruits and 6.8% (3.9% to 16.7%) for vegetables under a no deal Brexit scenario (S3), as shown in Supplementary Table 8. Incorporating cross-price elasticities between fresh and canned, dried, and frozen F&V in the model only slightly increased the effect of Brexit scenarios on intake of fresh F&V by up to 0.5% (Supplementary Table 9). This would result to approximately 12,990 (7,000 to 24,790) extra CVD deaths between 2021 and 2030 under a no deal Brexit scenario (S3) (Supplementary Table 10).

Our model that examined potential increases in the domestic production of some F&V did not substantially change our findings. The estimated effect on intake was 0.7% lower for fruits and 1.5% for vegetables in the last year of the modelling period compared to the first year, under a no deal Brexit scenario (S3) (Supplementary Table 11). Over the study period, this would contribute to between 210 and 720 fewer CVD deaths under the different scenarios compared to the main analysis for which domestic production was assumed to be stable (Supplementary Table 10).

DISCUSSION

This is the first study to exclusively quantify the potential impacts of post-Brexit trade scenarios on F&V prices, intake, and consequent CVD outcomes. Estimates from all modelled scenarios suggested that F&V prices would increase, hence reducing intake and contributing to additional CVD mortality. A no deal Brexit scenario could be the most harmful, increasing CHD and stroke deaths by approximately 0.9% (4,110 deaths) and 2.9% (8,290 deaths) respectively between 2021 and 2030. The least disruptive scenario modelled, which assumes a free trading agreement with the EU and half of non-EU F&V importers, could increase CHD and stroke deaths by approximately 0.3% (1,360 deaths) and 1.0% (2,740 deaths) respectively.

For peer review only - http://bmjopen.bmj.com/site/about/guidelines.xhtml

Our findings are consistent with previous preliminary analyses which suggest likely increases in F&V prices post-Brexit. A report from the House of Lords notes the vulnerability of F&V to price changes post-Brexit.[30] An empirical estimation showed that the cost of consuming five F&V per day as per governmental guidelines will increase by £2.20 a week for a household of four under a no deal Brexit scenario, after considering price inflation and an increase in import tariffs.[31] Analysis using a partial equilibrium model, which uses supply and demand curves to examine the effect of policy actions on single markets, has also shown that a no deal Brexit might increase prices of fruits by 3.1% and vegetables by 4.0% although disaggregated effects on F&V subgroups were not further investigated.[32] These analyses did not compare a no deal Brexit scenario with other potential post-Brexit trade options. Furthermore, they did not account for the potential effect of non-tariff trade barriers which are likely to be an important driver of price change post-Brexit.

This study has several strengths. It estimates the effect of different Brexit scenarios on the price of disaggregated F&V subgroups, taking into account both own- and cross-price effects to estimate changes in F&V intake. Sensitivity analysis also incorporated the additional effects of substitution with canned, dried, and frozen F&V. This study uses nationally representative data on F&V intake and purchases. It also employs CVD mortality projections using a Bayesian Age-Period-Cohort model which has been shown to produce more robust predictions compared with conventional methods.[33]

However, it also has some limitations. We used the most recent import data from 2015 to approximate F&V imports post-Brexit, assuming that F&V import shares will not change. This assumption might overestimate the price effects of scenarios that involve free trading agreements (S1 and S2) as the UK could shift towards importers that trade under these free trading agreements. There might be some discrepancies in the definition of F&V subgroups across different datasets. We explicitly describe each definition in Appendix A. Trade transaction costs due to technical regulations are based on published estimates derived using non-tariff measure data from 65 countries.[26] These estimates might overestimate the real costs as they might also reflect a price effect attributed to an increase in demand after complying with quality improving technical regulations in some countries. In this analysis, we conservatively omitted the potential impact of inflation on F&V price to minimise model uncertainty, which might underestimate price effects. We used robust estimates of UK specific price elasticities.[17] However, these elasticities were estimated using historical purchase data (2000-2009) and might not reflect the consumer response to price change after a potential wider economic shock of Brexit. Our model assumes a linear association between price, intake, and CVD outcomes under the different Brexit scenarios. However, real life associations in the medium and long-term may be characterised by feedback mechanisms in response to system

For peer review only - http://bmjopen.bmj.com/site/about/guidelines.xhtml

disruptions, such as Brexit. This can be addressed using computational general equilibrium models, which have been widely used to estimate Brexit effects on trade, productivity, and welfare[34, 35] but are more appropriate when modelling wider economic impacts across various sectors. Although, this study includes a sensitivity analysis that investigates a potential increase in domestic production of fruits and vegetables as a feedback response to an increase in price of imports, it does not provide a comprehensive investigation of the economic feedback effects of our modelled Brexit scenarios. Finally, we modelled four post-Brexit trade scenarios reflecting governmental and expert consensus.[36] However, ongoing negotiations within the British government and the Conservative party[37] and between the British government and the EU[38] means that the political space around Brexit is continuously changing and more trade policy options might arise.

F&V intake in the UK is suboptimal with less than half of the population consuming daily recommended levels.[11] Our analysis suggests that all modelled Brexit trade policy scenarios could increase price and reduce intake of F&V. The UK government needs to develop a comprehensive policy response against this potential detrimental impact by aligning domestic nutrition and agricultural policies and future trade policy with public health aims. At the same time, the wider implications of Brexit to the UK food system should also be considered. For example, approximately 80,000 seasonal workers are employed in the UK every year to harvest F&V, with the majority of them recruited from EU countries.[39] Abolishment of the free movement of labour guaranteed by EU regulations is likely to reduce available workforce for the horticulture sector with potential implications to prices of domestically produced F&V. Brexit may also affect food quality and safety in the UK by potentially relaxing risk prevention requirements, like the precautionary principle for food which is currently eliminated from the EU (Withdrawal) Act 2018,[40] in order to increase competitiveness in international markets. Moreover, Brexit is likely to affect prices and intake of other food groups with potential implications for health. For example, a previous analysis has shown that a no deal Brexit scenario would increase prices of all food products, with higher impacts on dairy, oils and fats, and meat.[32] Finally, the wider effects of Brexit on the UK economy could affect the whole food supply chain, including packaging, distribution, and retail of both fresh and processed foods. Future work should monitor and evaluate the population health implications of these potentially important changes in the UK food system.

In summary, post-Brexit trade policy could increase price and decrease intake of F&V, thus increasing CVD mortality in England. The UK government should therefore carefully consider the population health implications of Brexit during upcoming negotiations and post-Brexit planning, particularly adverse changes to food systems.

For peer review only - http://bmjopen.bmj.com/site/about/guidelines.xhtml

Contributor information: PS, AL, JPS, MOF, CM conceived the study. PS and PB analysed the data with inputs from JPS, BC, MGC, AL, MOF. PS drafted the paper with inputs from AL, CM, JPS, BC, MGC, SC, MOF. All authors made a substantial contribution to the data interpretation and critical review of the submitted manuscript.

Funding: PS, AL and CM are funded by the National Institute for Health Research (NIHR) via a Research Professorship Award to CM (grant number: RP 2014-04-032). NIHR had no role in the design, analysis or writing of this article. The Public Health Policy Evaluation Unit is grateful for the support of the NIHR School of Public Health Research.

Competing interests: None

Data sharing: Model input data are available on request from the corresponding author.

For peer review only

FIGURES

Figure 1. Estimated relative change in price of fruit and vegetable subgroups under each modelled Brexit scenario. Error bars indicate 95% uncertainty intervals.

Scenario S1: Free trading agreement with the EU and third countries; Scenario S2: Free trading agreement with the EU; Scenario S3: No deal Brexit; Scenario S4: Liberalised trade regime

Figure 2. Estimated number of cumulative CVD deaths for 2021-2030 associated with each modelled Brexit scenario, by sex.

Scenario S1: Free trading agreement with the EU and third countries; Scenario S2: Free trading agreement with the EU; Scenario S3: No deal Brexit; Scenario S4: Liberalised trade regime

For peer review only

REFERENCES

1. Schram A, Ruckert A, VanDuzer JA, et al. A conceptual framework for investigating the impacts of international trade and investment agreements on noncommunicable disease risk factors. *Health Policy Plan*. 2018;33(1):123-36.
2. Friel S, Hattersley L, Snowdon W, et al. Monitoring the impacts of trade agreements on food environments. *Obes Rev*. 2013;14 Suppl 1:120-34.
3. Stuckler D, McKee M, Ebrahim S, et al. Manufacturing Epidemics: The Role of Global Producers in Increased Consumption of Unhealthy Commodities Including Processed Foods, Alcohol, and Tobacco. *PLoS Med*. 2012;9(6):e1001235.
4. Baker P, Friel S. Food systems transformations, ultra-processed food markets and the nutrition transition in Asia. *Global Health*. 2016;12(1):80.
5. Thow AM, Hawkes C. The implications of trade liberalization for diet and health: a case study from Central America. *Global Health*. 2009;5(1):5.
6. Barlow P, McKee M, Basu S, et al. Impact of the North American Free Trade Agreement on high-fructose corn syrup supply in Canada: a natural experiment using synthetic control methods. *Cmaj*. 2017;189(26):E881-e7.
7. Department for Environment Food & Rural Affairs (DEFRA). Food statistics pocketbook 2017. 2017. Available from: https://www.gov.uk/government/statistics/food-statistics-pocketbook-2017. (accessed July 2018).
8. Department for Environment Food & Rural Affairs (DEFRA). Agriculture in the United Kingdom 2017.; 2018.
9. Farrow L, Georgieva I. Trade-offs in future food systems – consumer perspectives. A GFS Food Futures panel project. 2016.
10. Aune D, Giovannucci E, Boffetta P, et al. Fruit and vegetable intake and the risk of cardiovascular disease, total cancer and all-cause mortality—a systematic review and dose-response meta-analysis of prospective studies. *Int J Epidemiol*. 2017;46(3):1029-56.
11. Caireen Roberts, Toni Steer, Natalie Maplethorpe, et al. National Diet and Nutrition Survey Results from Years 7 and 8 (combined) of the Rolling Programme (2014/2015 to 2015/2016). Public Health England; 2018.
12. Allen K, Pearson-Stuttard J, Hooton W, et al. Potential of trans fats policies to reduce socioeconomic inequalities in mortality from coronary heart disease in England: cost effectiveness modelling study. *BMJ*. 2015;351:h4583.
13. Pearson-Stuttard J, Critchley J, Capewell S, et al. Quantifying the Socio-Economic Benefits of Reducing Industrial Dietary Trans Fats: Modelling Study. *PLoS One*. 2015;10(8):e0132524.
14. Collins M, Mason H, O'Flaherty M, et al. An Economic Evaluation of Salt Reduction Policies to Reduce Coronary Heart Disease in England: A Policy Modeling Study. *Value in Health*. 2014;17(5):517-24.
15. WTO. Tariff Analysis Online. Available from: http://tao.wto.org/default.aspx. (accessed February 2018).
16. HM Revenue & Customs. UK Trade Statistics. Available from: https://www.uktradeinfo.com/. (accessed February 2018).
17. Tiffin R, Balcombe K, Salois M, et al. Estimating Food and Drink Elasticities. 2011. Available from: https://www.gov.uk/government/uploads/system/uploads/attachment_data/file/137726/defra-stats-foodfarm-food-price-elasticities-120208.pdf. (accessed February 2017).
18. NatCen Social Research, MRC Elsie Widdowson Laboratory, University College London. Medical School. National Diet and Nutrition Survey Years 1-8, 2008/09-2015/16. 9th Edition ed: UK Data Service; 2018

For peer review only - http://bmjopen.bmj.com/site/about/guidelines.xhtml

19. Office for National Statistics, Department for Environment Food and Rural Affairs. Living Costs and Food Survey, 2016-2017. UK Data Service; 2018
20. Micha R, Peñalvo JL, Cudhea F, et al. Association between dietary factors and mortality from heart disease, stroke, and type 2 diabetes in the united states. *JAMA*. 2017;317(9):912-24.
21. Singh GM, Danaei G, Farzadfar F, et al. The Age-Specific Quantitative Effects of Metabolic Risk Factors on Cardiovascular Diseases and Diabetes: A Pooled Analysis. *PLoS One*. 2013;8(7):e65174.
22. Department for Environment Food & Rural Affairs (DEFRA). Horticulture Statistics 2016. . 2017. Available from: https://www.gov.uk/government/collections/horticultural-statistics. (accessed February 2017).
23. EU Customs Union. Available from: http://trade.ec.europa.eu/tradehelp/eu-customs-union. (accessed July 2018).
24. HM Government. The United Kingdom's exit from and new partnership with the European Union. 2017.
25. Abreu MD. Preferential rules of origin in regional trade agreements. World Trade Organization, Economic Research and Statistics Division; 2013.
26. Cadot O, Gourdon J. Non-tariff measures, preferential trade agreements, and prices: new evidence. *Rev World Econ*. 2016;152(2):227-49.
27. Pearson-Stuttard J, Bandosz P, Rehm CD, et al. Reducing US cardiovascular disease burden and disparities through national and targeted dietary policies: A modelling study. *PLoS Med*. 2017;14(6):e1002311.
28. Bajekal M, Scholes S, Love H, et al. Analysing Recent Socioeconomic Trends in Coronary Heart Disease Mortality in England, 2000-2007: A Population Modelling Study. *PLoS Med*. 2012;9(6):e1001237.
29. Capewell S, O'Flaherty M. Rapid mortality falls after risk-factor changes in populations. *The Lancet*. 2011;378(9793):752-3.
30. HOUSE OF LORDS European Union Committee. Brexit: food prices and availability. 2018.
31. The Food Foundation. FARMING FOR 5-A-DAY: Brexit Bounty or Dietary Disaster? ; 2017.
32. Clarke S, Serwicka I, Winters LA. Will Brexit Raise the Cost of Living? *National Institute Economic Review*. 2017;242(1):R37-R50.
33. Guzman Castillo M, Gillespie DOS, Allen K, et al. Future Declines of Coronary Heart Disease Mortality in England and Wales Could Counter the Burden of Population Ageing. *PLoS One*. 2014;9(6):e99482.
34. Sampson T. Brexit: The Economics of International Disintegration. *J Econ Perspect*. 2017;31(4):163-84.
35. Dhingra S, Huang H, Ottaviano G, et al. The Costs and Benefits of Leaving the EU: Trade Effects. *Centre for Economic Performance Technical Report*. 2016.
36. House of Commons International Trade Committee. . UK trade options beyond 2019. 2017.
37. HM Government. The future relationship between the United Kingdom and the European Union. 2018.
38. European Commission - Speech - Statement by Michel Barnier at the press conference following his meeting with Dominic Raab, UK Secretary of State for Exiting the EU [press release]. 2018.
39. NFU Access to a competent and flexible workforce. 2017.
40. European Union (Withdrawal) Act 2018. Available from: https://services.parliament.uk/bills/2017-19/europeanunionwithdrawal.html. (accessed July 2018).

For peer review only

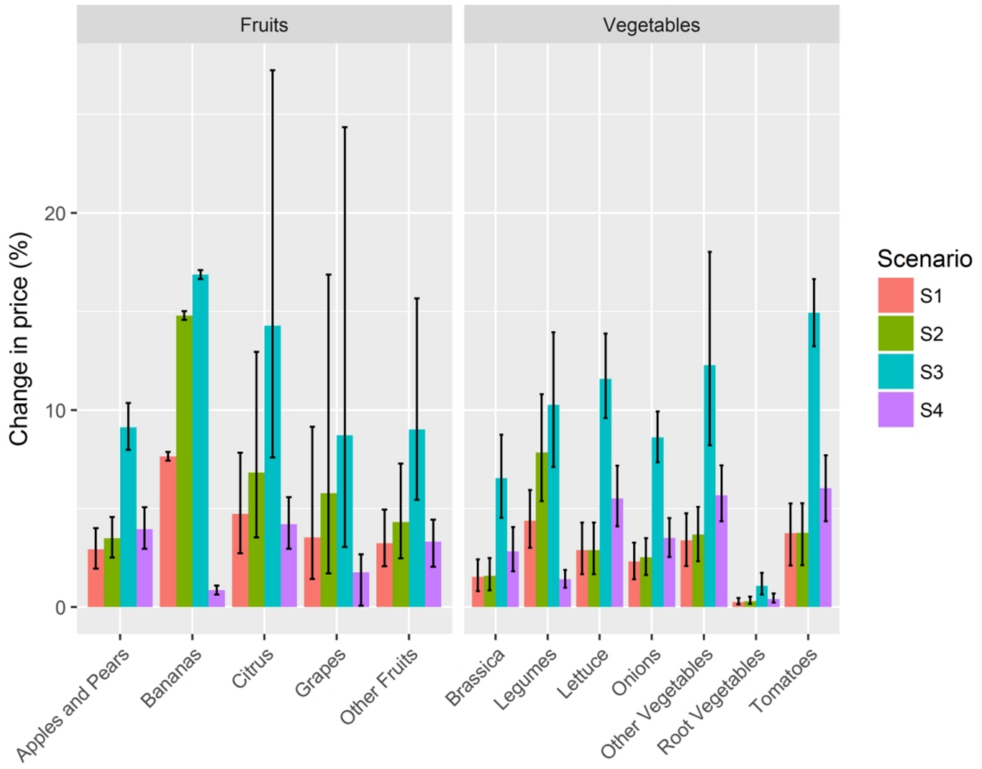

Figure 1. Estimated relative change in price of fruit and vegetable subgroups under each modelled Brexit scenario. Error bars indicate 95% uncertainty intervals.
Scenario S1: Free trading agreement with the EU and third countries; Scenario S2: Free trading agreement with the EU; Scenario S3: No deal Brexit; Scenario S4: Liberalised trade regime

129x105mm (300 x 300 DPI)

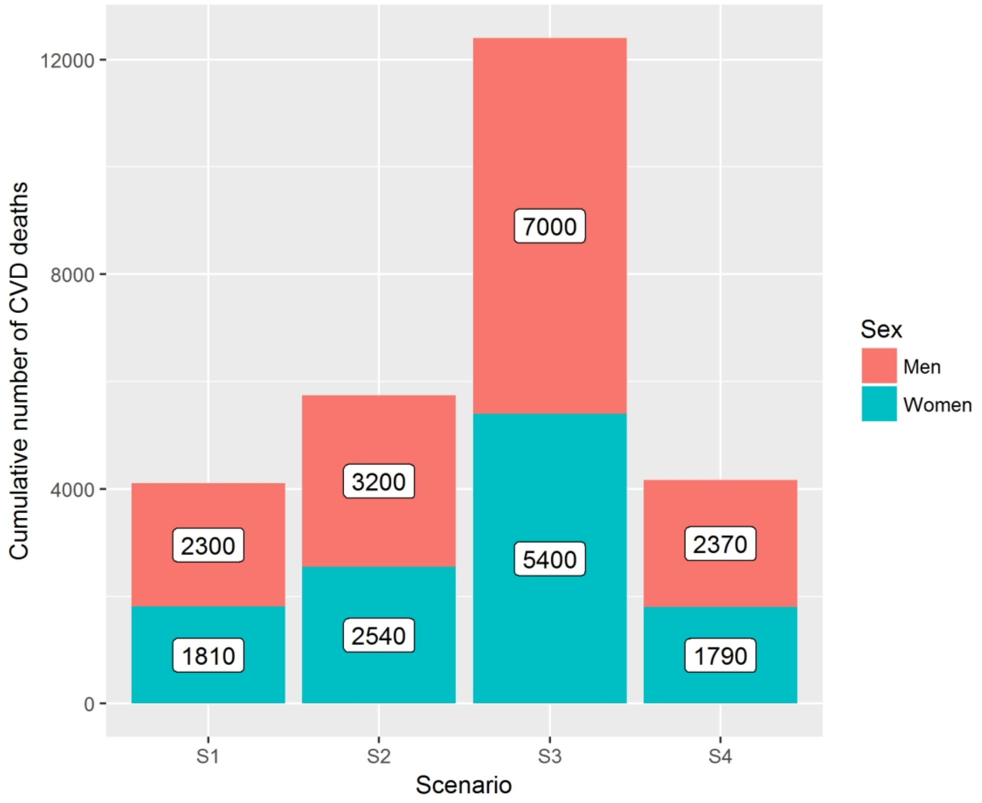

Figure 2. Estimated number of cumulative CVD deaths for 2021-2030 associated with each modelled Brexit scenario, by sex.
Scenario S1: Free trading agreement with the EU and third countries; Scenario S2: Free trading agreement with the EU; Scenario S3: No deal Brexit; Scenario S4: Liberalised trade regime

159x129mm (300 x 300 DPI)

Supplementary Appendix

Appendix A. Trade data inputs

The data inputs used in the policy layer of the model are presented in Table A1. These include fruit and vegetable (F&V) imports to the UK from the EU and from non-EU countries, either dutiable or under a duty-free arrangement, estimates of applied import tariffs, and estimates of imports as a percentage of overall F&V supply. We used a combination of data from the World Trade Organization (WTO),[1] the HM Revenues and Customs (HMRC),[2] and the Department for Environment, Food & Rural Affairs (DEFRA)[3] to obtain these estimates. The methodology used is described in detail below.

F&V were classified into five groups of fruits and seven groups of vegetables, in accordance to the classification applied by Tiffin et al. to estimate the price elasticities used in this model. We also identified two categories of canned, dried, and frozen F&V that were used in the sensitivity analysis. We used the Harmonised System (HS) 2012 classification to assign traded commodities to these groups. The HS has been developed by the World Customs Organization and has international applications, facilitating trade across participating countries. It uses a six-digit code system, arranged in three levels of disaggregation (two-digit, four-digit, and six-digit) to classify commodities. In this analysis, we used the four-digit HS level. The commodities included in each subgroup of F&V are presented in Table A2.

We used the latest available data (2015) from the HMRC[2] to identify imports of F&V (in net mass, kg) to the UK by country of origin for each F&V subgroup. Using information from the WTO, we identified the duty arrangement that is in place between each of the UK's F&V importers and the EU. These included bilateral or multilateral preferential arrangements or, if no agreement was in place, trade under the Most Favoured Nation (MFN) principle. The MFN principle prevents WTO members from discriminating between countries by imposing a common MFN duty to all their trading partners that are not part of a preferential trade agreement. We, then, identified which preferential arrangements allowed for duty-free trade for each F&V subgroup. As different duty rates are applied to the single products that comprise each commodity group (tariff lines), we defined as a duty-free arrangement every arrangement that gave duty-free access to at least 50% of tariff lines in each F&V subgroup.

We used data from the WTO[1] to estimate the mean ad valorem MFN duty applied by the EU and its standard deviation for each F&V subgroup. The WTO provides several averaging methods for applied tariffs in each commodity group. We used the HS sub-heading averaging method which gives equal importance to all HS sub-headings included in each commodity group.[1] The standard

For peer review only - http://bmjopen.bmj.com/site/about/guidelines.xhtml

deviation shows the dispersion of HS sub-heading averages around the overall simple average of the commodity group. This method is not biased by the different levels of disaggregation in the applied tariffs across WTO members, allowing for a uniform comparison across countries. When more than one commodity code was included in our F&V subgroups (i.e. for other and canned, dried, and frozen F&V), we used the same method to average across groups by summing the average tariff of each commodity code and dividing by the total number of subheadings included in the group. The combined standard deviation for these groups was estimated using the following formula:

$$Combined\ SD = \frac{\sqrt{\sum(n*(s^2+(\bar{x}-\bar{X})^2)}}{\sum n}$$

where n is the number of subheadings, s the standard deviation for each subheading, $\bar{x}$ the average tariff for each subheading, and $\bar{X}$ the average tariff of all subheadings.

Tariffs for all F&V subgroups were ad valorem with the exception of tomatoes which have a specific duty defined in €/100 kg. The World Bank provides estimations of the ad valorem equivalent of specific duties.[4] These varied based on the declared price of tomatoes. We conservatively used the smaller tariff provided, as tomato price is seasonal and fluctuates significantly thoughtout the year.

Finally, we estimated F&V imports as a percentage of F&V supply. DEFRA provides information on imports, exports, and production of F&V.[3] Total supply was defined as the sum of domestic production and imports minus exports. We estimated imports as a percentage of total supply for years 2006 to 2016 to capture annual variations at the medium term. We then used the mean and standard deviation of these estimates for our model. We assigned the F&V products provided by DEFRA into our F&V subgroups as shown in Table A2.

For peer review only - http://bmjopen.bmj.com/site/about/guidelines.xhtml

Table A1. Trade related inputs of the model policy layer.

	Imports from the EU (% of total imports)	Duty-free imports from non-EU countries (% of total imports)	Dutiable imports from non-EU countries (% of total imports)	Ad valorem EU MFN duty (%) Mean (std dev)	Imports (% of supply) Mean (std dev)
Fruits					
Apples and Pears	67.1	21.5	11.4	7.2 (0.0)	71.5 (2.4)
Bananas	10.2	89.2	0.6	16.0 (0.0)	100.0 (0)
Citrus fruit	51.2	43.1	5.7	10.8 (5.5)	100.0 (0)
Grapes	26.2	63.8	10.1	8.4 (6.0)	100.0 (0)
Other Fruits	49.2	38.2	12.6	7.0 (3.4)	87.0 (1.2)
Vegetables					
Brassica	97.0	2.7	0.3	12.0 (0.0)	31.0 (4.6)
Legumes	18.7	78.5	2.8	9.6 (1.6)	92.6 (8.2)
Lettuce	99.8	0.1	0.0	10.4 (0.0)	58.8 (4.1)
Onions	82.4	8.5	9.2	10.0 (0.4)	50.9 (2.5)
Other vegetables	88.5	9.0	2.5	9.8 (3.5)	70.6 (2.1)
Root vegetables	86.2	13.4	0.3	13.3 (0.3)	5.1 (1.3)
Tomatoes	88.6	0.3	11.2	10.8 (0.0)	82.8 (1.2)
Canned, dried, and frozen					
Fruits	61.7	22.6	15.7	8.1 (5.2)	87.0 (1.2)
Vegetables	62.5	7.2	30.3	7.3 (6.8)	70.6 (2.1)

Table A2. Classification of F&V across different data sources.

	Trade and Tariff data (HS 4-digit classification)	Supply data (DEFRA)	Living Costs and Food Survey – household purchases
Fruits			
Apples and Pears	0808 - Apples, pears and quinces, fresh	Total Dessert Apples; Total Culinary Apples; Total Pears	Fresh apples, Fresh pears
Bananas	0803 - Bananas, incl. plantains, fresh or dried	Bananas	Fresh bananas
Citrus fruit	0805 - Citrus fruit, fresh or dried	Citrus	Fresh oranges, Other fresh citrus fruits
Grapes	0806 - Grapes, fresh or dried	Grapes	Fresh grapes

For peer review only

Other Fruits	0804 - Dates, figs, pineapples, avocados, guavas, mangoes and mangosteens, fresh or dried; 0807 - Melons, incl. watermelons, and papaws "papayas", fresh; 0809 - Apricots, cherries, peaches incl. nectarines, plums and sloes, fresh; 0810 - Fresh strawberries, raspberries, blackberries, back, white or red currants, gooseberries and other edible fruits	Total Plums; Cherries; Others & Mixed; Strawberries; Raspberries; Blackcurrants; Other Soft Fruit	Fresh stone fruit, Other fresh soft fruit, Fresh melons, Other fresh fruit
Vegetables			
Brassica	0704 - Cabbages, cauliflowers, kohlrabi, kale and similar edible brassicas, fresh or chilled	Cauliflowers and Broccoli; Cabbages; Other Brassicas	Fresh cabbages, Fresh brussels sprouts, Fresh cauliflower
Legumes	0708 - Leguminous vegetables, shelled or unshelled, fresh or chilled	Beans; Peas; Dried Peas; Dried Chick Peas; Dried Beans	Fresh peas, Fresh beans
Lettuce	0705 - Lettuce "Lactuca sativa" and chicory "Cichorium spp.", fresh or chilled	Lettuce; Lettuce - protected veg	Leafy salads fresh (Lettuce and leafy salads, Prepared lettuce salads)
Onions	0703 - Onions, shallots, garlic, leeks and other alliaceous vegetables, fresh or chilled	Onions, Dry Bulb; Onions, Spring; Leeks; Garlic; Other Alliaceous; Onions	Fresh onions, leeks and shallots
Other vegetables	0707 - Cucumbers and gherkins, fresh or chilled; 0709 - Other vegetables, fresh or chilled	Asparagus; Baby leaf; Celery; Courgette; Cucumbers; Mushrooms; Others-protected veg; Others, field grown; Rhubarb; Self Blanching Celery; Sweet Peppers; Watercress; All Other Dried Vegetables; All Other Fresh Vegetables; Aubergines	Other fresh green vegetables, Fresh cucumbers, Fresh mushrooms, Miscellaneous fresh vegetables
Root vegetables	0706 - Carrots, turnips, salad beetroot, salsify, celeriac,	Beetroot; Carrots; Parsnips; Turnips and	Fresh carrots, Fresh turnips and swede, Other fresh root vegetables

For peer review only - http://bmjopen.bmj.com/site/about/guidelines.xhtml

	radishes and similar edible roots, fresh or chilled	Swedes; Carrots and Turnips	
Tomatoes	0702 - Tomatoes, fresh or chilled	Tomatoes	Fresh tomatoes
	Canned, dried, and frozen		
Fruits	0811 - Fruit and nuts, uncooked or cooked by steaming or boiling in water, frozen, whether or not containing added sugar or other sweetening matter; 0813 - Dried apricots, prunes, apples, peaches, pears, papaws "papayas", tamarinds and other edible fruits, and mixtures of edible and dried fruits or of edible nuts; 0814 - Peel of citrus fruit or melons, incl. watermelons, fresh, frozen, dried or provisionally preserved in brine, or in water with other additives	Approximated from "Other Fruits"	Tinned peaches, pears and pineapples; All other tinned or bottled fruit; Dried fruit; Frozen strawberries, apple slices, peach halves, oranges and other frozen fruits
Vegetables	0710 - Vegetables, uncooked or cooked by steaming or boiling in water, frozen; 0712 - Dried vegetables, whole, cut, sliced, broken or in powder, but not further prepared 0713 - Dried leguminous vegetables, shelled, whether or not skinned or split	Approximated from "Other Vegetables"	Tomatoes, canned or bottled; Peas, canned; Beans canned; Other canned vegetables; Dried pulses other than air-dried; Air-dried vegetables; Peas, frozen; Beans, frozen; Other frozen vegetables

For peer review only

For peer review only - http://bmjopen.bmj.com/site/about/guidelines.xhtml

Appendix B. Mortality projections.

We used a Bayesian Age-Period-Cohort (BAPC) model to estimate mortality projections between 2021 and 2030. The BAPC model attributes changes in mortality to (1) age effects, which capture physiological and social changes related to aging; (2) period effects, which are associated with environmental, scientific, and social changes related to calendar time of death; and (3) cohort effects, which describe generational differences due to year of birth. It then projects mortality probabilities by assuming that trends of these effects continue over time. The methodology has been previously described in detail.[5] Briefly, we used historic CHD and stroke mortality data stratified by sex and age (10-year groups from 25-34 until 85+) between 1981 and 2015 from the Office for National Statistics (ONS), defined using ICD-codes (Table B1). We also used historic population estimates (1985-2015) and population projections (2016-2030) by age and sex from the ONS. We applied the model using the BAMP statistical software which employs Markov Chain Monte Carlo simulations to estimate mortality projections. An age and sex specific ischemic to haemorrhagic stroke ratio (Table B2), estimated using 2016 mortality data by ICD-code from the ONS, was applied to overall stroke projections to estimate ischemic and haemorrhagic stroke mortality between 2021 and 2030, assuming no changes in clinical care over time. We combined ischemic and not specified stroke to better reflect evidence from the American Heart Association that suggests 87% of all stroke is ischemic.[6] Previous analyses in the UK have followed the same approach.[7]

Table B1. ICD codes for CVD outcomes

Type of CVD outcomes	ICD-9 codes (1981-2000)	ICD-10 codes (2011-2015 and 2016)
CHD	410-414	I20-I25
Overall stroke	430-438	I60-I69
Ischemic stroke		I63, I65-I67 (except I67.4)
Haemorrhagic stroke		I60-I62, I69.0-I69.2, I67.4
Other (not specified) stroke		I64, I69.4, I69.8

For peer review only - http://bmjopen.bmj.com/site/about/guidelines.xhtml

Table B2. Percentage of stroke deaths attributed to ischemic, haemorrhagic, and other (not specified) stroke and ischemic to haemorrhagic stroke ratio

Population group	Ischemic stroke	Haemorrhagic stroke	Other (not specified) stroke	Ischemic: Haemorrhagic*
Men 25-34	14%	82%	5%	0.22
Men 35-44	12%	79%	9%	0.26
Men 45-54	17%	67%	16%	0.50
Men 55-64	18%	53%	30%	0.90
Men 65-74	23%	37%	40%	1.71
Men 75-84	24%	28%	48%	2.57
Men 85+	30%	19%	51%	4.25
Women 25-34	23%	69%	9%	0.46
Women 35-44	14%	81%	5%	0.23
Women 45-54	14%	73%	13%	0.38
Women 55-64	15%	63%	23%	0.59
Women 65-74	18%	46%	36%	1.16
Women 75-84	23%	32%	46%	2.13
Women 85+	30%	17%	53%	4.98
Overall	*26%*	*28%*	*46%*	*2.58*

*Other (not specified) stroke was combined with ischemic stroke.

For peer review only

For peer review only - http://bmjopen.bmj.com/site/about/guidelines.xhtml

Appendix C. Effect of Brexit scenarios on price of F&V

Under each scenario, we assumed two main drivers of F&V price change. The first driver is the application of tariffs to imported commodities based on their country of origin. If there is a free trading agreement in place, these tariffs will be zero. However, if trade occurs under WTO regulations, the UK will have to apply import tariffs. These are required to be equal among all trading partners under the WTO's MFN clause that protects its members from discriminatory treatment. We assumed that the UK will adopt the EU MFN tariffs for all scenarios except scenario 4 (Liberalised regime) when the UK will reduce its MFN tariffs to zero.

The second driver of price change is attributed to an increase in transaction costs of EU trade due to post-Brexit border controls. We assumed that border controls will occur (1) to apply rules of origin and (2) to check technical requirements, such as Technical Barriers to Trade (TBT) and Sanitary and Phytosanitary (SPS) measures. Rules of origin determine commodities' country of origin and are necessary in order to apply appropriate trade restrictions such as tariffs. Within the Customs Union, such restrictions do not apply and goods move across borders freely. However, exiting the EU Customs Union will require checks for rules of origin for all commodities entering the UK from the EU. The cost of ensuring compliance with rules of origin regulations varies between different countries, depending on their trade arrangements, their infrastructure, the product traded, and other factors[8]. A WTO Working Paper[8] suggests that administrative costs of compliance with rules of origin vary between 2% and 8%, recommending a 5% estimate as a commonly used benchmark.

In common with other free trading arrangements, the EU has integrated technical regulations that do not require border controls. However, when no free trading agreement is in place and trade occurs under WTO regulations, technical characteristics of commodities need to be checked at the border. Cadot et al. investigated the difference between the price-raising effect of SPS and TBT by commodity in the presence and absence of preferential arrangements that involve deep integration of technical requirements[9]. They estimated a difference of 4.5% for vegetable products, which we used to approximate the increase in transaction costs of F&V under the Brexit scenarios that no preferential arrangement is assumed between the UK and the EU (Scenario 3 – No deal Brexit and Scenario 4 – Liberalised regime). However, this might overestimate the real effect as the analysis by Cadot et al. does not specify if the price-raising effects of SPS and TBT are due to transaction costs or demand increases reflecting the quality improvements that usually follow compliance with technical requirements.

For peer review only - http://bmjopen.bmj.com/site/about/guidelines.xhtml

Appendix D. Estimating the effect of F&V intake change on mortality

The IMPACT Food Policy model estimates the proportional change in mortality due to change in the intake of each F&V subgroup by age and sex using the following formula:

$$MortalityChange = (1 - e^{beta \times IntakeChange})$$

where *beta* is the natural logarithm of the relative risk between fruit or vegetable intake and CVD outcomes and *IntakeChange* is the change in intake of each F&V subgroup under the different scenarios.

The number of deaths attributable to each scenario for every F&V subgroup by age, sex, and year are then estimated as shown below:

$$Deaths = MortalityChange * Mortality$$

where *Mortality* is the counterfactual number of predicted deaths of CHD, ischemic stroke or haemorrhagic in each age-sex group in years 2021 to 2030.

In order to combine the effect of the different F&V subgroups into an overall F&V effect we used the cumulative risk-reduction approach as previously described by Bajekal et al.[10] Briefly, we estimated an adjustment factor as the ratio of an additive and a multiplicative effect as shown below:

$$AF = CR/AR$$

where

$$CR = 1 - \big(1 - abs(MortalityChange_1)\big) * \big(1 - abs(MortalityChange_2)\big) * \ldots * \big(1 - abs(MortalityChange_i)\big)$$

and

$$AR = abs(MortalityChange_1) + abs(MortalityChange_2) + \cdots + abs(MortalityChange_i)$$

with *1, 2, …, i* being the different F&V subgroups.

We then estimated the overall attributable deaths as shown in the following equation

$$Deaths_{overall} = (Deaths_1 + Deaths_2 + \cdots + Deaths_i) * AF$$

where $Deaths_1$, $Deaths_2$, …, $Deaths_i$ are the number of deaths due to change in intake of each F&V subgroup.

For peer review only - http://bmjopen.bmj.com/site/about/guidelines.xhtml

Appendix E. Sensitivity analysis – substitution with canned, dried, and frozen F&V

We performed a sensitivity analysis in order to take into account substitution between fresh F&V and canned, dried, and frozen F&V. Trade data inputs for canned, dried, and frozen F&V and their classification using commodity codes are presented in Tables A1 and A2 respectively. Intake of canned, dried, and frozen F&V was estimated by applying a gradient of purchases from the Living Costs and Food Survey[11] to the overall F&V intake, similarly to F&V subgroups estimation (Table A2). We used own and cross-price elasticities for "Fresh fruits", "Fresh vegetables", "Tinned & dried fruit", and "Canned vegetables" by Tiffin et al. Frozen F&V were not included in the estimation of price elasticities.

For the model calculations, we first estimated the cross-price effect of the change in the price of fresh F&V on canned, dried, and frozen F&V using the appropriate cross-price elasticity. The effect of the different Brexit scenarios on price of overall fresh F&V was calculated as the ratio of the final changes in fresh F&V intake (after substitution between F&V subgroups was taken into account) to the own-price elasticities of fresh fruits and fresh vegetables.

We, then, estimated the effect of the different Brexit scenarios on prices of canned, dried, and frozen F&V, as described in Appendix B. Own and cross-price elasticities were employed to estimate the effect of the change in the price of canned, dried, and frozen F&V on the intake of canned, dried, and frozen F&V (own-price effect) and fresh F&V (cross-price effect), after the new equilibrium was reached. The overall intake change was the difference between baseline intake and intake when both prices of fresh F&V and canned, dried, and frozen F&V had changed. Effect of change in intake of fresh F&V on mortality was estimated as described in Appendix C.

For peer review only

Supplementary tables

Supplementary Table 1. Relative risks of the association between F&V intake and CHD, ischemic stroke and haemorrhagic stroke.

	RR per serving of fruit intake			RR per serving of vegetable intake		
Age group	CHD	Ischemic stroke	Haemorrhagic stroke	CHD	Ischemic stroke	Haemorrhagic stroke
25-34	0.92 (0.87, 0.97)	0.83 (0.76, 0.9)	0.63 (0.49, 0.81)	0.93 (0.89, 0.97)	0.76 (0.64, 0.9)	0.76 (0.61, 0.95)
35-44	0.92 (0.87, 0.97)	0.83 (0.77, 0.9)	0.64 (0.5, 0.82)	0.93 (0.9, 0.97)	0.77 (0.66, 0.9)	0.77 (0.62, 0.95)
45-54	0.93 (0.89, 0.97)	0.86 (0.8, 0.92)	0.69 (0.56, 0.84)	0.94 (0.91, 0.97)	0.80 (0.7, 0.92)	0.80 (0.67, 0.96)
55-64	0.94 (0.91, 0.98)	0.88 (0.83, 0.93)	0.73 (0.61, 0.87)	0.95 (0.93, 0.98)	0.83 (0.74, 0.93)	0.83 (0.72, 0.96)
65-74	0.95 (0.92, 0.98)	0.90 (0.86, 0.94)	0.77 (0.67, 0.89)	0.96 (0.94, 0.98)	0.86 (0.78, 0.94)	0.86 (0.76, 0.97)
75+	0.97 (0.96, 0.99)	0.94 (0.92, 0.96)	0.86 (0.8, 0.92)	0.98 (0.97, 0.99)	0.92 (0.87, 0.96)	0.92 (0.86, 0.97)

Source: Micha et al, 2017[12]

For peer review only

For peer review only - http://bmjopen.bmj.com/site/about/guidelines.xhtml

Supplementary Table 2. Statistical distributions for model inputs and corresponding parameters used in the probabilistic sensitivity analysis.

Inputs	Distribution	Parameters	Source
Ad valorem EU MFN tariff (%)	Beta	Alpha=((1-mean)/variance - 1/mean)*mean^2; beta=alpha*(1/mean - 1) estimated from tariff averages and variance	Tariff Analysis Online, WTO[1]
Imports % supply	Beta	Alpha=((1-mean)/variance - 1/mean)*mean^2; beta=alpha*(1/mean - 1) estimated from the mean and variance of imports between 2006-2016	DEFRA[3]
Cost of rules of origin	Pert	mode: 5%; min: 2%; max: 8%	Abreu, 2013[8]
Cost of technical regulations	Pert	mode: 4.5%, max/min: +/-20%	Cadot, 2016[9]
Mortality	Pert	mode: death projections, best estimate; min: death projections, lower 95% credible limit; max: death projections, upper 95% credible limit	Own estimations using ONS data
Elasticities	Normal	Mean and SE using price elasticities between 2000 and 2009	Tiffin, 2011[13]
F&V intake	Gamma	alpha: mean^2/variance; beta: variance/mean	National Diet and Nutrition Survey Rolling Programme[14]
Relative Risks	Log normal	Parameters based on Barendregt, 2010[15]	Micha, 2017[12]

For peer review only - http://bmjopen.bmj.com/site/about/guidelines.xhtml

Supplementary Table 3. Estimated relative change in price of fruit and vegetable subgroups under each modelled Brexit scenario. Numbers in parentheses indicate 95% uncertainty intervals.

	Scenario 1	Scenario 2	Scenario 3	Scenario 4
Fruits				
Apples and Pears	2.9% (2.0%, 4.0%)	3.5% (2.5%, 4.6%)	9.1% (8.0%, 10.4%)	4.0% (3.0%, 5.1%)
Bananas	7.6% (7.4%, 7.9%)	14.8% (14.6%, 15.0%)	16.9% (16.6%, 17.1%)	0.9% (0.6%, 1.1%)
Citrus fruit	4.7% (2.7%, 7.8%)	6.8% (3.5%, 12.9%)	14.3% (7.6%, 27.2%)	4.2% (3.0%, 5.6%)
Grapes	3.5% (1.4%, 9.1%)	5.8% (1.7%, 16.9%)	8.7% (3.1%, 24.3%)	1.8% (0.1%, 2.7%)
Other fruits	3.2% (2.1%, 4.9%)	4.3% (2.5%, 7.3%)	9.0% (5.4%, 15.7%)	3.3% (2.1%, 4.4%)
Vegetables				
Brassica	1.5% (0.8%, 2.4%)	1.6% (0.8%, 2.5%)	6.5% (4.5%, 8.7%)	2.8% (1.8%, 4.1%)
Legumes	4.4% (3.0%, 5.9%)	7.8% (5.4%, 10.8%)	10.3% (7.1%, 13.9%)	1.4% (1.0%, 1.9%)
Lettuce	2.9% (1.7%, 4.3%)	2.9% (1.7%, 4.3%)	11.6% (9.6%, 13.9%)	5.5% (4.1%, 7.2%)
Onions	2.3% (1.4%, 3.3%)	2.5% (1.6%, 3.5%)	8.6% (7.4%, 9.9%)	3.5% (2.5%, 4.5%)
Other vegetables	3.4% (2.1%, 4.8%)	3.7% (2.3%, 5.1%)	12.3% (8.2%, 18.0%)	5.7% (4.3%, 7.2%)
Root vegetables	0.3% (0.1%, 0.5%)	0.3% (0.2%, 0.5%)	1.1% (0.6%, 1.7%)	0.4% (0.2%, 0.7%)
Tomatoes	3.8% (2.1%, 5.3%)	3.8% (2.1%, 5.3%)	14.9% (13.2%, 16.6%)	6.0% (4.4%, 7.7%)

Supplementary Table 4. Mean (standard deviation) intake of F&V (g/d) in England by age and sex, estimated using data from the National Diet and Nutrition Survey Rolling Programme Years 1-8.

Population group	Fruit intake	Vegetable intake
Men 25-44	86.5 (95.3)	187.9 (99.3)
Men 45-64	116.6 (114.0)	200.7 (109.0)
Men 65+	121.6 (111.8)	184.6 (101.8)
Women 25-44	93.3 (102.1)	184.8 (111.6)
Women 45-64	128.5 (118.1)	202.0 (115.0)
Women 65+	132.2 (117.1)	173.6 (86.3)
Overall	*111.1 (111.1)*	*190.0 (106.1)*

Means and standard deviation are weighted for non-response

For peer review only - http://bmjopen.bmj.com/site/about/guidelines.xhtml

For peer review only

Supplementary Table 5. Gradient of F&V subgroups intake, estimated using household purchase data (g/person/week) from the Family Food module of the Living Costs and Food Survey 2016/7.

F&V subgroup	Intake (%)
Apples & Pears	19.1
Bananas	25.5
Citrus fruit	16.1
Grapes	6.8
Other fruits	26.9
Canned, dried, and frozen fruits	*5.5*
Total fruits	***100.0***
Brassica	9.4
Legumes	2.0
Lettuce	4.5
Onions	10.4
Other vegetables	24.6
Root vegetables (excl. potatoes)	16.6
Tomatoes	7.7
Canned, dried, and frozen vegetables	*24.8*
Total vegetables	***100.0***

For peer review only

For peer review only - http://bmjopen.bmj.com/site/about/guidelines.xhtml

Supplementary Table 6. Estimated relative change in intake of F&V subgroups under each modelled Brexit scenario. Numbers in parentheses indicate 95% uncertainty intervals.

	Scenario 1	Scenario 2	Scenario 3	Scenario 4
Fruits	***-4.4%<br>(-5.2%, -3.8%)***	***-7.0%<br>(-8.4%, -5.9%)***	***-11.4%<br>(-14.2%, -9.5%)***	***-2.8%<br>(-3.3%, -2.3%)***
Apples and Pears	-3.3%<br>(-4.1%, -2.5%)	-4.4%<br>(-5.4%, -3.5%)	-9.4%<br>(-10.7%, -8.2%)	-3.5%<br>(-4.3%, -2.7%)
Bananas	-5.4%<br>(-5.8%, -5.0%)	-10%<br>(-10.8%, -9.3%)	-12.1%<br>(-13.2%, -11.2%)	-1.1%<br>(-1.4%, -0.9%)
Citrus fruit	-4.4%<br>(-7.1%, -2.7%)	-6.4%<br>(-11.6%, -3.6%)	-13.0%<br>(-24%, -7.3%)	-3.8%<br>(-5%, -2.7%)
Grapes	-3.3%<br>(-6.7%, -2.0%)	-5.4%<br>(-11.9%, -3.0%)	-8.2%<br>(-17.3%, -4.9%)	-1.8%<br>(-2.4%, -0.7%)
Other fruits	-4.3%<br>(-5.7%, -3.3%)	-6.2%<br>(-8.7%, -4.7%)	-11.4%<br>(-16.8%, -8.3%)	-3.6%<br>(-4.6%, -2.5%)
Vegetables	***-2.5%<br>(-3.1%, -1.9%)***	***-2.7%<br>(-3.3%, -2.2%)***	***-9.1%<br>(-11.0%, -7.8%)***	***-4.0%<br>(-4.6%, -3.4%)***
Brassica	-1.5%<br>(-2.2%, -1.0%)	-1.5%<br>(-2.2%, -0.9%)	-6.4%<br>(-8.1%, -4.9%)	-2.8%<br>(-3.7%, -2.0%)
Legumes	-3.3%<br>(-4.5%, -2.3%)	-5.7%<br>(-7.8%, -4.0%)	-8.3%<br>(-11%, -6.1%)	-1.6%<br>(-2.1%, -1.1%)
Lettuce	-2.7%<br>(-3.7%, -1.8%)	-2.9%<br>(-3.9%, -2.0%)	-10.2%<br>(-11.8%, -8.7%)	-4.6%<br>(-5.9%, -3.6%)
Onions	-2.2%<br>(-3%, -1.5%)	-2.4%<br>(-3.2%, -1.7%)	-8.3%<br>(-9.5%, -7.3%)	-3.5%<br>(-4.3%, -2.7%)
Other vegetables	-3.7%<br>(-5.0%, -2.6%)	-4.1%<br>(-5.5%, -2.9%)	-13.4%<br>(-18.6%, -9.7%)	-6.1%<br>(-7.5%, -4.9%)
Root vegetables	-0.9%<br>(-1.1%, -0.6%)	-0.9%<br>(-1.2%, -0.6%)	-3.5%<br>(-4.2%, -2.8%)	-1.5%<br>(-1.8%, -1.2%)
Tomatoes	-3%<br>(-4.1%, -1.9%)	-3.1%<br>(-4.2%, -2%)	-11.8%<br>(-13.1%, -10.6%)	-4.8%<br>(-5.9%, -3.6%)

For peer review only

For peer review only - http://bmjopen.bmj.com/site/about/guidelines.xhtml

Supplementary Table 7. Estimated number of counterfactual (95% credible interval) and attributable (95% uncertainty interval) CHD and stroke deaths and mortality increase (%) in 2030, by age and sex, under each modelled Brexit scenario.

		Scenario 1	Scenario 2	Scenario 3	Scenario 4
CVD	Counterfactual deaths (2030)	71120 (38140, 164210)	71120 (38140, 164210)	71120 (38140, 164210)	71120 (38140, 164210)
	Attributable deaths (2030)	450 (250, 810)	630 (340, 1,180)	1,340 (780, 2,350)	450 (270, 750)
	Mortality reduction (%)	0.6%	0.9%	1.9%	0.6%
CHD	Counterfactual deaths (2030)	43110 (27160, 73740)	43110 (27160, 73740)	43110 (27160, 73740)	43110 (27160, 73740)
	Attributable deaths (2030)	140 (80, 230)	200 (110, 340)	420 (260, 690)	140 (90, 210)
	Mortality reduction (%)	0.3%	0.5%	1.0%	0.3%
Stroke	Counterfactual deaths (2030)	28010 (10970, 90470)	28010 (10970, 90470)	28010 (10970, 90470)	28010 (10970, 90470)
	Attributable deaths (2030)	320 (180, 590)	440 (240, 860)	960 (550, 1720)	330 (190, 570)
	Mortality reduction (%)	1.1%	1.5%	3.3%	1.1%

For peer review only

For peer review only - http://bmjopen.bmj.com/site/about/guidelines.xhtml

Supplementary Table 8. Estimated relative change in price of fresh and canned, dried, and frozen F&V under each modelled Brexit scenario. Numbers in parentheses indicate 95% uncertainty intervals.

	Scenario 1	Scenario 2	Scenario 3	Scenario 4
Fresh fruits	4.4%<br>(3.8%, 5.2%)	7.1%<br>(6%, 8.5%)	11.6%<br>(9.6%, 14.4%)	2.9%<br>(2.4%, 3.3%)
Canned, dried, and frozen fruits	3.4%<br>(2.1%, 5%)	4.1%<br>(2.4%, 6.9%)	10.3%<br>(5.7%, 20.0%)	4%<br>(2%, 5.6%)
Fresh vegetables	2.5%<br>(2%, 3%)	2.7%<br>(2.2%, 3.3%)	9.2%<br>(7.8%, 11%)	4%<br>(3.4%, 4.6%)
Canned, dried, and frozen vegetables	2.4%<br>(1.4%, 3.3%)	2.6%<br>(1.5%, 3.9%)	6.8%<br>(3.9%, 16.7%)	3.1%<br>(-1.2%, 4.8%)

Supplementary Table 9. Estimated relative change in intake of fresh and canned, dried, and frozen F&V under each modelled Brexit scenario. Numbers in parentheses indicate 95% uncertainty intervals.

	Scenario 1	Scenario 2	Scenario 3	Scenario 4
Fresh fruits	-4.5%<br>(-5.3%, -3.8%)	-7.1%<br>(-8.5%, -6.0%)	-11.7%<br>(-14.4%, -9.7%)	-2.9%<br>(-3.4%, -2.4%)
Canned, dried, and frozen fruits	-2.4%<br>(-3.7%, -1.3%)	-2.8%<br>(-5.0%, -1.3%)	-7.3%<br>(-15.3%, -3.8%)	-3.0%<br>(-4.2%, -1.4%)
Fresh vegetables	-2.6%<br>(-3.2%, -2.1%)	-2.9%<br>(-3.5%, -2.3%)	-9.6%<br>(-11.4%, -8.2%)	-4.1%<br>(-4.8%, -3.6%)
Canned, dried, and frozen vegetables	-1.0%<br>(-1.6%, -0.4%)	-1.1%<br>(-1.9%, -0.4%)	-2.6%<br>(-8.5%, -0.7%)	-1.2%<br>(-2.3%, 1.4%)

For peer review only

For peer review only - http://bmjopen.bmj.com/site/about/guidelines.xhtml

Supplementary Table 10. Estimated number of cumulative CVD deaths and 95% uncertainty intervals for 2021-2030 associated with each modelled Brexit scenario – Sensitivity analyses

Scenario	Coronary heart disease	Stroke	Cardiovascular disease
Sensitivity Analysis A: Taking into account effect of canned, dried, and frozen F&V			
Scenario 1	1,400 (760, 2,830)	2,860 (1,440, 5,590)	4,260 (2,200, 8,420)
Scenario 2	1,970 (1,010, 4,190)	3,980 (1,940, 8,170)	5,950 (2,940, 12,360)
Scenario 3	4,230 (2,370, 8,070)	8,760 (4,630, 16,720)	12,990 (7,000, 24,790)
Scenario 4	1,390 (830, 2,420)	2,920 (1,610, 5,200)	4,310 (2,430, 7,620)
Sensitivity Analysis B: Increasing domestic production by 2% a year*			
Scenario 1	1,300 (690, 2,550)	2,590 (1,310, 5,190)	3,890 (2,000, 7,730)
Scenario 2	1,850 (930, 3,860)	3,650 (1,790, 7,620)	5,500 (2,720, 11,470)
Scenario 3	3,890 (2,190, 7,300)	7,800 (4,090, 14,880)	11,680 (6,280, 22,180)
Scenario 4	1,260 (740, 2,150)	2,590 (1,420, 4,700)	3,860 (2,150, 6,850)

**Increase in domestic supply was assumed for all F&V excluding bananas, citrus fruits, and grapes.*

Supplementary Table 11. Estimated relative change in intake of F&V in the first year (2021) and last year (2030) of the modelling period under each modelled Brexit scenario, assuming that domestic supply of F&V will increase by approximately 2% a year*. Numbers in parentheses indicate 95% uncertainty intervals.

Scenario	Change in intake	
	Fruits	Vegetables
2021		
Scenario 1	-4.4% (-5.2%, -3.8%)	-2.5% (-3.0%, -1.9%)
Scenario 2	-7.0% (-8.4%, -5.9%)	-2.7% (-3.3%, -2.2%)
Scenario 3	-11.4% (-14.2%, -9.5%)	-9.1% (-11.0%, -7.8%)
Scenario 4	-2.8% (-3.3%, -2.3%)	-4.0% (-4.6%, -3.4%)
2030		
Scenario 1	-4.1% (-4.9%, -3.5%)	-2.1% (-2.5%, -1.6%)
Scenario 2	-6.6% (-8.0%, -5.7%)	-2.3% (-2.7%, -1.8%)
Scenario 3	-10.7% (-13.3%, -8.9%)	-7.6% (-9.2%, -6.5%)
Scenario 4	-2.5% (-2.9%, -2.1%)	-3.3% (-3.8%, -2.8%)

**Increase in domestic supply was assumed for all F&V excluding bananas, citrus fruits, and grapes.*

For peer review only

For peer review only - http://bmjopen.bmj.com/site/about/guidelines.xhtml

Supplementary figures

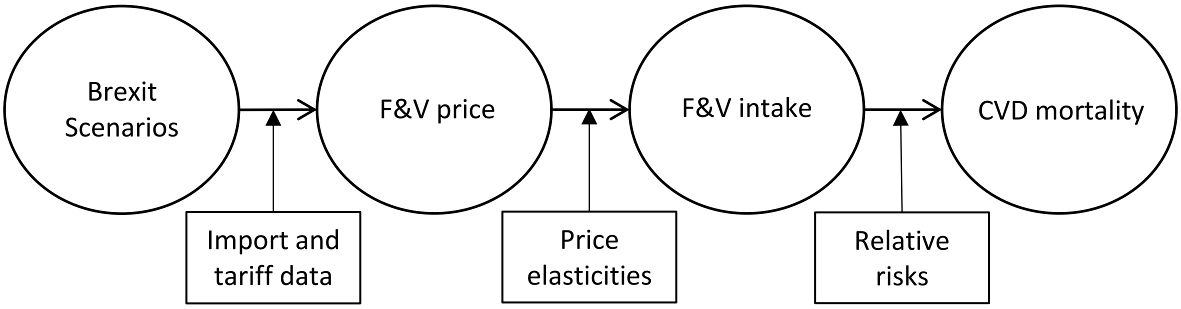

Supplementary Figure 1. Schematic representation of the model and its main inputs.

References

1. WTO. Tariff Analysis Online. Available from: http://tao.wto.org/default.aspx. (accessed February 2018).

2. HM Revenue & Customs. UK Trade Statistics. Available from: https://www.uktradeinfo.com/. (accessed February 2018).

3. Department for Environment Food & Rural Affairs (DEFRA). Horticulture Statistics 2016. . 2017. Available from: https://www.gov.uk/government/collections/horticultural-statistics. (accessed February 2017).

4. World Bank World Integrated Trade Solution (WITS). Available from: https://wits.worldbank.org/default.aspx. (accessed April 2018).

5. Guzman Castillo M, Gillespie DOS, Allen K, et al. Future Declines of Coronary Heart Disease Mortality in England and Wales Could Counter the Burden of Population Ageing. *PLoS One*. 2014;9(6):e99482.

6. Benjamin EJ, Virani SS, Callaway CW, et al. Heart Disease and Stroke Statistics—2018 Update: A Report From the American Heart Association. *Circulation*. 2018.

7. Brownrigg JRW, Hughes CO, Burleigh D, et al. Microvascular disease and risk of cardiovascular events among individuals with type 2 diabetes: a population-level cohort study. *The Lancet Diabetes & Endocrinology*. 2016;4(7):588-97.

8. Abreu MD. Preferential rules of origin in regional trade agreements. World Trade Organization, Economic Research and Statistics Division; 2013.

9. Cadot O, Gourdon J. Non-tariff measures, preferential trade agreements, and prices: new evidence. *Rev World Econ*. 2016;152(2):227-49.

10. Bajekal M, Scholes S, Love H, et al. Analysing Recent Socioeconomic Trends in Coronary Heart Disease Mortality in England, 2000-2007: A Population Modelling Study. *PLoS Med*. 2012;9(6):e1001237.

11. Office for National Statistics, Department for Environment Food and Rural Affairs. Living Costs and Food Survey, 2016-2017. UK Data Service; 2018

12. Micha R, Peñalvo JL, Cudhea F, et al. Association between dietary factors and mortality from heart disease, stroke, and type 2 diabetes in the united states. *JAMA*. 2017;317(9):912-24.

For peer review only

For peer review only - http://bmjopen.bmj.com/site/about/guidelines.xhtml

13. Tiffin R, Balcombe K, Salois M, et al. Estimating Food and Drink Elasticities. 2011. Available from: https://www.gov.uk/government/uploads/system/uploads/attachment_data/file/137726/defra-stats-foodfarm-food-price-elasticities-120208.pdf. (accessed February 2017).

14. NatCen Social Research, MRC Elsie Widdowson Laboratory, University College London. Medical School. National Diet and Nutrition Survey Years 1-8, 2008/09-2015/16. 9th Edition ed: UK Data Service; 2018

15. Barendregt JJ. The effect size in uncertainty analysis. *Value Health*. 2010;13(4):388-91.

For peer review only

STROBE Statement—checklist of items that should be included in reports of observational studies

	Item No	Recommendation	
**Title and abstract**	1	(*a*) Indicate the study's design with a commonly used term in the title or the abstract	Study design indicated in title and abstract, p.1 and 2-3
		(*b*) Provide in the abstract an informative and balanced summary of what was done and what was found	Summary provided in the abstract, p.2-3
**Introduction**			
Background/rationale	2	Explain the scientific background and rationale for the investigation being reported	Background and rational explained, p.4
Objectives	3	State specific objectives, including any prespecified hypotheses	Specific objective stated, p.4
**Methods**			
Study design	4	Present key elements of study design early in the paper	Key elements of modelling analysis presented, p.4
Setting	5	Describe the setting, locations, and relevant dates, including periods of recruitment, exposure, follow-up, and data collection	Location and dates are specified, p.4
Participants	6	(*a*) *Cohort study*—Give the eligibility criteria, and the sources and methods of selection of participants. Describe methods of follow-up *Case-control study*—Give the eligibility criteria, and the sources and methods of case ascertainment and control selection. Give the rationale for the choice of cases and controls *Cross-sectional study*—Give the eligibility criteria, and the sources and methods of selection of participants	Cross-sectional studies for data inputs: Eligibility criteria and sources of selection stated, p.5
		(*b*) *Cohort study*—For matched studies, give matching criteria and number of exposed and unexposed *Case-control study*—For matched studies, give matching criteria and the number of controls per case	Not applicable
Variables	7	Clearly define all outcomes, exposures, predictors, potential confounders, and effect modifiers. Give diagnostic criteria, if applicable	Outcomes, exposures, and confounders/effect modifiers are defined, p.5-6 and 8
Data sources/ measurement	8*	For each variable of interest, give sources of data and details of methods of assessment (measurement). Describe comparability of assessment methods if there is more than one group	Data sources and assessment methods are described, p. 5-6
Bias	9	Describe any efforts to address potential sources of bias	Age and sex stratification was performed to address potential bias, p. 5

For peer review only - http://bmjopen.bmj.com/site/about/guidelines.xhtml

Study size	10	Explain how the study size was arrived at	Not applicable
Quantitative variables	11	Explain how quantitative variables were handled in the analyses. If applicable, describe which groupings were chosen and why	Analysis of quantitative variables described, p.8 and in more detail in the Appendix
Statistical methods	12	(*a*) Describe all statistical methods, including those used to control for confounding	All statistical methods described, p.8-9 and in more detail in the Appendix
		(*b*) Describe any methods used to examine subgroups and interactions	Subgroup analysis, i.e. main analysis, is described, p.8 and in more detail in the Appendix
		(*c*) Explain how missing data were addressed	Not applicable
		(*d*) *Cohort study*—If applicable, explain how loss to follow-up was addressed<br>*Case-control study*—If applicable, explain how matching of cases and controls was addressed<br>*Cross-sectional study*—If applicable, describe analytical methods taking account of sampling strategy	Not applicable
		(*e*) Describe any sensitivity analyses	Sensitivity analyses described in detail, p.9

Continued on next page

For peer review only

**Results**			
Participants	13*	(a) Report numbers of individuals at each stage of study—eg numbers potentially eligible, examined for eligibility, confirmed eligible, included in the study, completing follow-up, and analysed	Not applicable
		(b) Give reasons for non-participation at each stage	Not applicable
		(c) Consider use of a flow diagram	
Descriptive data	14*	(a) Give characteristics of study participants (eg demographic, clinical, social) and information on exposures and potential confounders	Baseline characteristics provided, p.10 and tables in the Appendix
		(b) Indicate number of participants with missing data for each variable of interest	Not applicable
		(c) *Cohort study*—Summarise follow-up time (eg, average and total amount)	Not applicable
Outcome data	15*	*Cohort study*—Report numbers of outcome events or summary measures over time	Number of outcome events over the modelling period reported, p.10-11
		Case-control study—Report numbers in each exposure category, or summary measures of exposure	
		Cross-sectional study—Report numbers of outcome events or summary measures	
Main results	16	(*a*) Give unadjusted estimates and, if applicable, confounder-adjusted estimates and their precision (eg, 95% confidence interval). Make clear which confounders were adjusted for and why they were included	Main estimates with 95% uncertainty intervals stated, p.9-11
		(*b*) Report category boundaries when continuous variables were categorized	Age categories defined, p. 6
		(*c*) If relevant, consider translating estimates of relative risk into absolute risk for a meaningful time period	Not applicable
Other analyses	17	Report other analyses done—eg analyses of subgroups and interactions, and sensitivity analyses	Results for sensitivity analysis reported, p. 11
**Discussion**			
Key results	18	Summarise key results with reference to study objectives	Key results summarised, p.11
Limitations	19	Discuss limitations of the study, taking into account sources of potential bias or imprecision. Discuss both direction and magnitude of any potential bias	Study limitations are described, p. 12-13
Interpretation	20	Give a cautious overall interpretation of results considering objectives, limitations, multiplicity of analyses, results from similar studies, and other relevant evidence	Results are cautiously interpreted throughout the discussion, p. 11-13
Generalisability	21	Discuss the generalisability (external validity) of the study results	Not applicable
**Other information**			
Funding	22	Give the source of funding and the role of the funders for the present study and, if applicable, for the original study on which the present article is based	Funding is declared

For peer review only - http://bmjopen.bmj.com/site/about/guidelines.xhtml
